# Pulse arrival time as a surrogate of blood pressure

**DOI:** 10.1038/s41598-021-01358-4

**Published:** 2021-11-23

**Authors:** Eoin Finnegan, Shaun Davidson, Mirae Harford, João Jorge, Peter Watkinson, Duncan Young, Lionel Tarassenko, Mauricio Villarroel

**Affiliations:** 1 Department of Engineering Science, Institute of Biomedical Engineering, University of Oxford, Oxford, UK; 2 Critical Care Research Group, Nuffield Department of Clinical Neurosciences, University of Oxford, Oxford, UK; 3 NIHR Oxford Biomedical Research Centre, Oxford, UK

**Keywords:** Cardiovascular biology, Cardiovascular diseases, Cardiology, Cardiovascular biology, Data processing

## Abstract

Various models have been proposed for the estimation of blood pressure (BP) from pulse transit time (PTT). PTT is defined as the time delay of the pressure wave, produced by left ventricular contraction, measured between a proximal and a distal site along the arterial tree. Most researchers, when they measure the time difference between the peak of the R-wave in the electrocardiogram signal (corresponding to left ventricular depolarisation) and a fiducial point in the photoplethysmogram waveform (as measured by a pulse oximeter attached to the fingertip), describe this erroneously as the PTT. In fact, this is the pulse arrival time (PAT), which includes not only PTT, but also the time delay between the electrical depolarisation of the heart’s left ventricle and the opening of the aortic valve, known as pre-ejection period (PEP). PEP has been suggested to present a significant limitation to BP estimation using PAT. This work investigates the impact of PEP on PAT, leading to a discussion on the best models for BP estimation using PAT or PTT. We conducted a clinical study involving 30 healthy volunteers (53.3% female, 30.9 ± 9.35 years old, with a body mass index of 22.7 ± 3.2 kg/m$^{2}$). Each session lasted on average 27.9 ± 0.6 min and BP was varied by an infusion of phenylephrine (a medication that causes venous and arterial vasoconstriction). We introduced new processing steps for the analysis of PAT and PEP signals. Various population-based models (Poon, Gesche and Fung) and a posteriori models (inverse linear, inverse squared and logarithm) for estimation of BP from PTT or PAT were evaluated. Across the cohort, PEP was found to increase by 5.5 ms ± 4.5 ms from its baseline value. Variations in PTT were significantly larger in amplitude, − 16.8 ms ± 7.5 ms. We suggest, therefore, that for infusions of phenylephrine, the contribution of PEP on PAT can be neglected. All population-based models produced large BP estimation errors, suggesting that they are insufficient for modelling the complex pathways relating changes in PTT or PAT to changes in BP. Although PAT is inversely correlated with systolic blood pressure (SBP), the gradient of this relationship varies significantly from individual to individual, from − 2946 to − 470.64 mmHg/s in our dataset. For the a posteriori inverse squared model, the root mean squared errors (RMSE) for systolic and diastolic blood pressure (DBP) estimation from PAT were 5.49 mmHg and 3.82 mmHg, respectively. The RMSEs for SBP and DBP estimation by PTT were 4.51 mmHg and 3.53 mmHg, respectively. These models take into account individual calibration curves required for accurate blood pressure estimation. The best performing population-based model (Poon) reported error values around double that of the a posteriori inverse squared model, and so the use of population-based models is not justified.

## Introduction

Hypertension (high blood pressure) has been shown to be one of the highest risk factors for the majority of cardiovascular diseases, including coronary heart disease, atrial fibrillation and stroke^1^. In addition, chronic hypertension has been shown to be an important modifiable risk factor for neurodegenerative diseases such as Alzheimer’s disease, Parkinson’s disease and dementia^2,3^. In a cross-sectional study of 153,996 adults spanning urban and rural areas across 17 countries, Chow et al.^4^ found that despite the high risks of cardiovascular disease, hypertension had a diagnosis rate of only 46%. Additionally, only a third of the individuals diagnosed with hypertension were able to keep their blood pressure (BP) levels under control.

Monitoring changes of BP in an ambulatory setting has been shown to be a stronger indicator of hypertension compared to spot measurements recorded in the clinic^5^. Ambulatory blood pressure monitoring (ABPM) is often performed over periods between 24 and 48 h, with BP readings taken using oscillometry, BP being measured every 30 min during the day and every hour at night by increasing and gradually releasing the pressure of an inflatable cuff applied to the arm^6^. These measurements are often noisy, uncomfortable and can disrupt sleep. In a study by Viera et al.^7^ across 60 adults, cuff inflation caused sleep arousal in over 70% of volunteers, and approximately a third of all volunteers reported bruising from the cuff. In addition, Wax et al.^8^ studied the differences between BP values measured by the cuff and an arterial line, used as a gold standard. While the mean error between the two devices was low (1 mmHg), the standard deviation of the errors was as high as 16 mmHg. To facilitate the assessment of hypertension in an ambulatory setting, new methods and devices are required for the non-invasive estimation of BP that that ideally would avoid the need for a cuff.

Recent advances in non-invasive estimation of BP have been focussed on the pulse transit time (PTT), defined as the time it takes for the pressure wave produced by left ventricular contraction to travel from one arterial site to another. PTT is typically computed as the time delay between two waveforms at a proximal and a distal location along the arterial tree^9^. Changes in PTT, or its counterpart pulse wave velocity (PWV), are shown via the Moens–Korteweg equation^10^ to be non-linearly dependent on arterial stiffness. As Gavish et al.^11^ show, arterial stiffness is not constant but rather a function of (among other things) BP, age and level of autonomic nervous system (ANS) activity. In a purely mechanical sense, as arteries stretch, more collagen fibres are recruited, leading to a non-linear (often thought to be exponential^12^) relationship between BP and arterial stiffness. Additionally, as an individual ages, elastin is replaced by collagen leading to arteriosclerosis, the thickening and hardening of the arterial walls. Together with these mechanical effects, arterial stiffness is also actively modulated by smooth muscle (which is found more prominently in peripheral arteries^9^), however, typically this component is assumed to be negligible^9^.

Many papers in the literature describe methods for BP estimation using PTT. Several studies induce changes in BP by artificial means to obtain a range of reference BP values to develop and validate their proposed methods. The most common method found in the literature is to vary the reference BP values using some form of exercise^9,13^. However, exercise causes motion artefacts and the physiological response to exercise will vary significantly from person to person. Other common methods for varying reference BP values are posture changes and mental stress, which reduce the occurence of motion artefacts. However, the changes in BP induced by these techniques are much lower than for exercise-induced changes^9^. There have been a few studies that have changed BP by medication infusion^14,15^. This has the advantage of achieving a wide range of BP values with minimal motion artefacts. As the relationship between changes in BP and changes in the velocity of the pressure wave depends on many parameters unique to an individual (such as arterial compliance, average cross-sectional area of the arteries and the distance between the two arterial sites), models require calibration to approximate this unique relationship. Various models have been proposed in the literature, with calibration typically requiring the approximation of two parameters: a slope and an intercept. This can be achieved by least squares approximation with two or more pairs of BP and PTT readings. To allow for real time estimation of BP, Poon et al.^16^, Chen et al.^17^ and Gesche et al.^18^ have proposed quick calibration by a single reference BP value (often recorded using a cuff) with the remaining parameters approximated based on population averages and physiological constants. However, the full utility of these models to encapsulate the unique and complex relationship between PTT and BP, remains unclear. A summary of BP estimation methods can be found in Refs.^9,13,19^.

Most researchers, when they measure the time difference between the peak of the R-wave in the electrocardiogram (ECG) signal (corresponding to left ventricular depolarisation) and a fiducial point in the photoplethysmogram (PPG) waveform (as measured by a pulse oximeter fingertip), describe this erroneously as the PTT. That is in fact the pulse arrival time (PAT), which includes not only the PTT, but also the time delay between electrical depolarisation of the heart’s left ventricle and ejection of blood through the aorta^20^, known as the pre-ejection period (PEP); i.e. ${\text{PTT}} = {\text{PAT}} - {\text{PEP}}$. PEP can vary depending on contractility, sympathetic nervous system activity, preload and afterload^21^. Exercise, medication and posture changes have all been shown to contribute significantly to changes in PEP^14,21–24^. Only a handful of publications have systematically studied the effect that PEP has on PAT estimates. Payne et al.^14^ studied changes in PAT and PEP across 12 healthy volunteers under an infusion of various medications including: Glyceryl trinitrate (GTN), Angiotensin, Norepinephrine and Salbutamol. Full details of PAT or PEP computation were not provided. However, the authors concluded that changes in PEP contributed a significant factor (12–35%) to the estimation of changes in PAT. Zhang et al.^21^ conducted a similar study in 6 anaesthetised dogs through the use of various medications. Estimates of systolic blood pressure (SBP) using PTT had a root mean squared error (RMSE) value of 5.3 mmHg, whereas estimates of SBP using PAT had a mean RMSE of 9.8 mmHg. However, it is unclear the extent to which this effect would translate to human subjects. By contrast, Wong et al.^22^ suggested that PAT can allow for more accurate tracking of BP, especially during periods where PEP is shown to be proportional to changes in BP such as post exercise. However, due to the opposing forces of preload and afterload, PEP is not always correlated with BP.

Despite the unknown contribution of PEP, PAT offers a good alternative for non-invasive monitoring of BP. ECG and PPG are readily available in the hospital, thus allowing for the development of BP estimation algorithms using PAT on large open source publicly available hospital databases^25–28^. Zheng et al.^29^ developed a wearable armband, capable of recording simultaneous ECG and PPG waveforms for PAT estimation. The device was tested on 10 healthy volunteers for 24 h each with BP recordings taken every 30 min. An RMSE value of BP estimation of 6.2 mmHg was reported. These two main factors have resulted in the large majority of research on non-invasive BP monitoring focussing on PAT. Other proximal waveforms have been proposed as alternatives to the ECG, such as the ballistocardiogram (BCG)^30–32^ and the impedance cardiogram (ICG)^33^. While these waveforms remove the impact of PEP, they often require specialist equipment and use signals that are more vulnerable to noise than the ECG^34^.

The pathways relating changes in BP to changes in PAT are complex. This work investigates the relationship between PAT, PEP and PTT to BP in a clinical study involving 30 healthy volunteers. Changes in BP were induced by an infusion of phenylephrine, a medication that stimulates alpha-1 receptors in blood vessels to cause them to constrict (vasoconstriction)^35^. BP values were measured using a cuff inflating every minute. We propose new processing steps for the PAT, PEP and PTT time series and discuss the impact of PEP on PAT. Additionally, we compare various models relating PAT and PTT to BP and highlight the most accurate model.

## Results

30 volunteers were recruited for our clinical study. The data from three volunteers was discarded from the analysis because the reference ECG waveform did not include any periods of high-quality data as a result of errors in the connection of the ECG electrodes. The demographics of the 27 participants in the study whose data was used for analysis are shown in Table 1. All volunteers were healthy, with a mean body mass index (BMI) of 22.9 kg/m$^{2}$ and no history of cardiovascular disease. On average, each session lasted 28.4 min. The average age of the volunteers was 29.7 years and there was an even gender split (51.9% female).

**Table 1:** Demographics of the population in the clinical study.

Descriptor	Value
Total number of volunteers	27
Average length of a session (min)$^{\mathrm{a}}$	27.9 (± 0.6)
Age (years)$^{\mathrm{a}}$	30.2 (± 8.2)
Gender (females)$^{\mathrm{b}}$	14 (51.9%)
Height (cm)$^{\mathrm{a}}$	170.6 (± 9.8)
Weight (kg)$^{\mathrm{a}}$	66.8 (± 12.4)
Body mass index (kg/m$^{2}$)$^{\mathrm{a}}$	22.8 (± 3.3)

$^{\mathrm{a}}$Mean (± standard deviation).

$^{\mathrm{b}}$N (% of total population).

**Figure 1:**
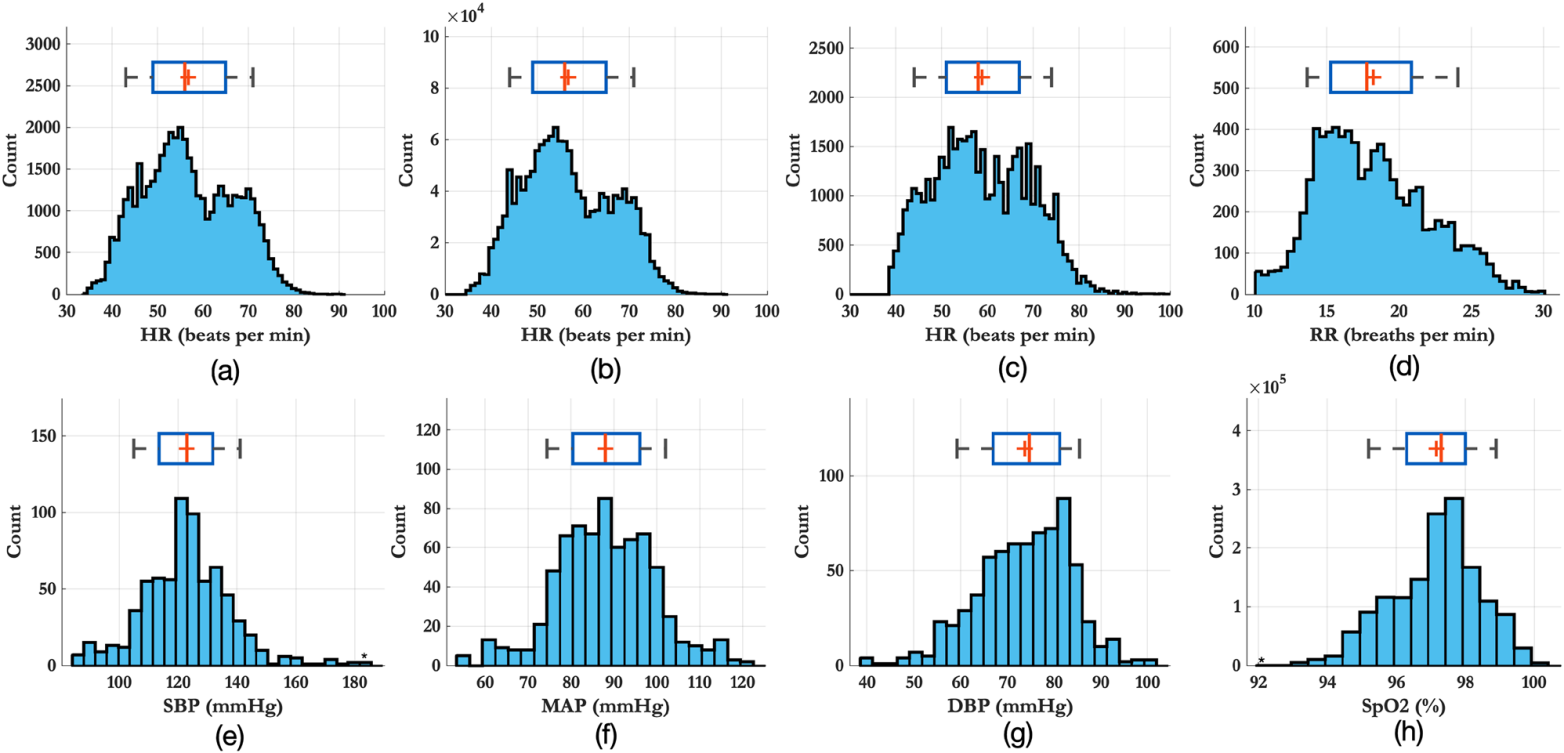
Distribution of physiological values recorded across the entire study in our dataset for all the volunteers: (**a**) heart rate from the Philips monitor, (**b**) heart rate from the Stowood device, (**c**) heart rate from the CardioScreen device, (**d**) respiratory rate estimated from the nasal cannula, (**e**) SBP from the Philips BP cuff, (**f**) MAP from the Philips BP cuff and (**g**) DBP from the Philips BP cuff, (**h**) $SpO_{2}$ from the Philips monitor.

**Figure 2:**
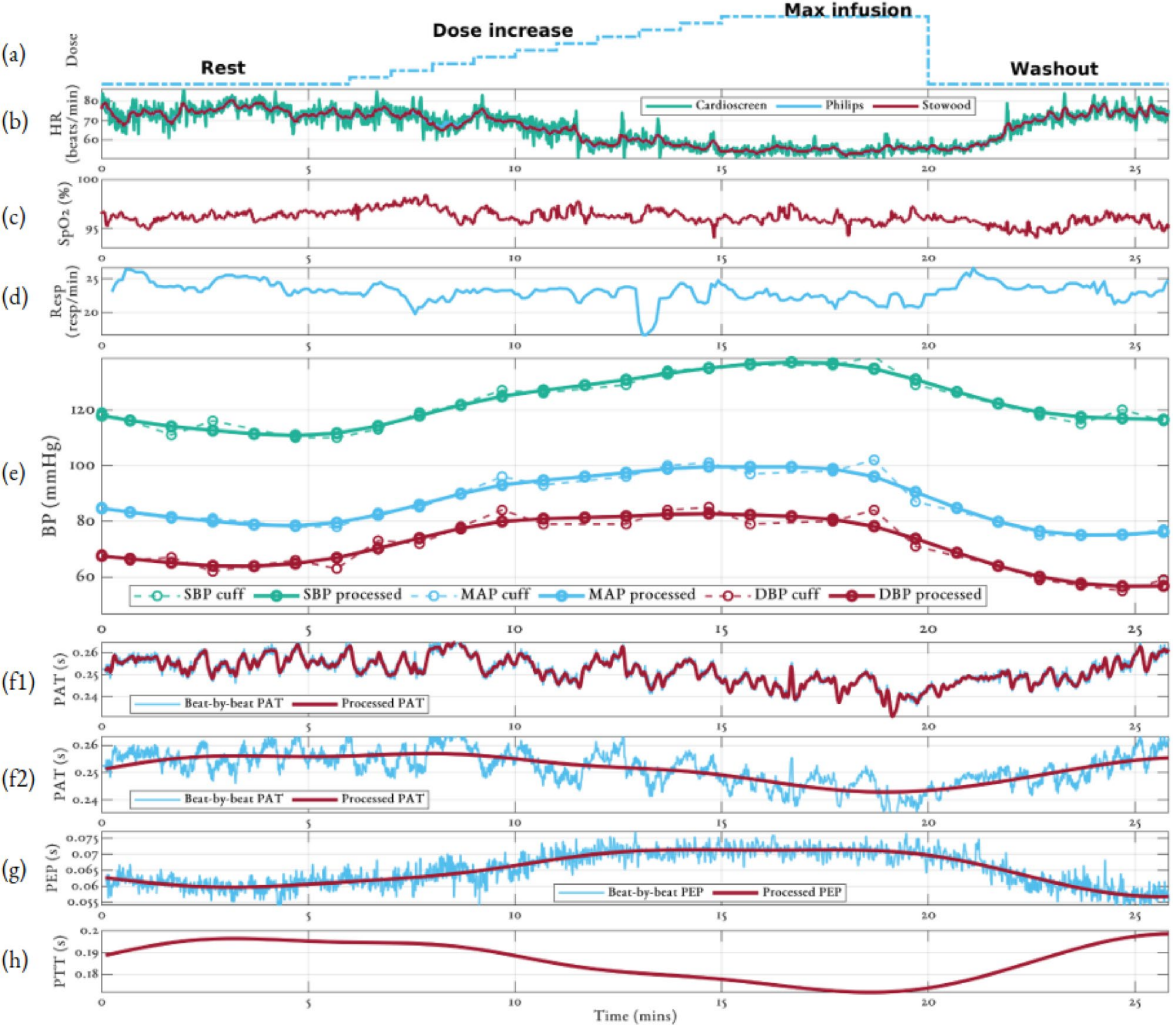
Physiological response for a 28 y/o female with BMI of 21.0. (**a**) Dose of phenylephrine. (**b**) Heart rate from the Cardioscreen, Philips and Stowood reference devices. (**c**) $SpO_{2}$ from the Stowood device, (**d**) respiratory rate from the nasal cannula, (**e**) BP from the cuff (dotted lines) and processed by our proposed algorithms (solid line), (**f1**) beat-by-beat PAT and filtered PAT with respiratory component removed, (**f2**) beat-by-beat PAT and processed PAT to compare to cuff values, (**g**) beat-by-beat PEP time series and processed PEP, and (**h**) PTT time series.

The distributions of the vital-signs recorded across the entire study for all 27 volunteers are shown in Fig. 1. Figure 2 illustrates the typical response to the phenylephrine dose for a 28-year-old female with a BMI of 21.0. Each session began with a 5-min resting period during which the volunteer’s vital signs were stable. Phenylephrine was infused at a rate predetermined by the volunteer’s weight with peak infusion occurring 14 min into the study. The maximum dose was maintained for a further 6 min. The session ended with an 8-min washout period. Peripheral oxygen saturation ($SpO_{2}$) and respiratory rate (RR) were typically stable throughout the study. Figure 2e shows the systolic blood pressure (SBP), mean arterial blood pressure (MAP) and diastolic blood pressure (DBP) values recorded using the cuff together with the BP signal computed by cubic splines (see Methods).

Supplementary material A provides an analysis of the frequency spectrum of PAT for this study. This highlighted three frequency bands of interest: a component less 0.083 Hz (the Nyquist limit of the BP cuff) representing the slow changes in BP induced by the phenylephrine infusion; a component of unknown origin between 0.083 and 0.1 Hz, which may be due to oscillations in BP caused by Mayer waves^36^; and a respiratory component between 0.17 and 0.35 Hz. Figure 2f1 shows beat-by-beat PAT and PAT low-pass filtered (0.15 Hz cut-off frequency) to remove the respiratory component and highlight the oscillations likely caused by the Mayer waves. Figure 2f2 shows the PAT signal after low-pass filtering to remove the Mayer wave component and compare to the BP cuff. Figure 2g shows the beat-by-beat PEP signal which has been processed to remove the small noise variation. The processed PAT and PEP estimates, for the volunteer shown in Fig. 2, show a decrease and increase respectively, in line with the changes in BP. (h) shows the PTT time series which is computed as the difference between the processed PAT and PEP time series.

### Individual correlation of PAT, PTT and PEP to BP

**Figure 3:**
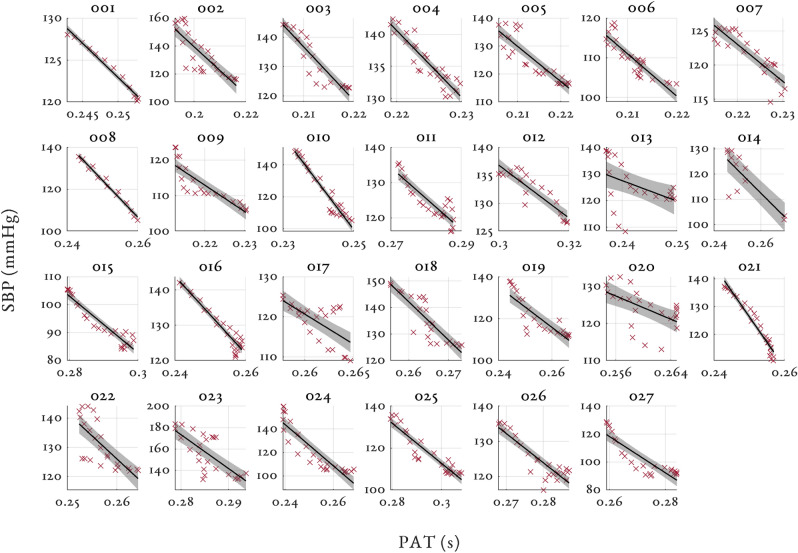
Individual relationships between SBP (mmHg) and PAT (s) for all volunteers in the study. The 95% confidence intervals are shown by the shaded region for each volunteer. Please note the large variations in the range of BP values for the y-axis (80 mmHg for subject 024, and 10 mmHg for subject 008).

**Figure 4:**
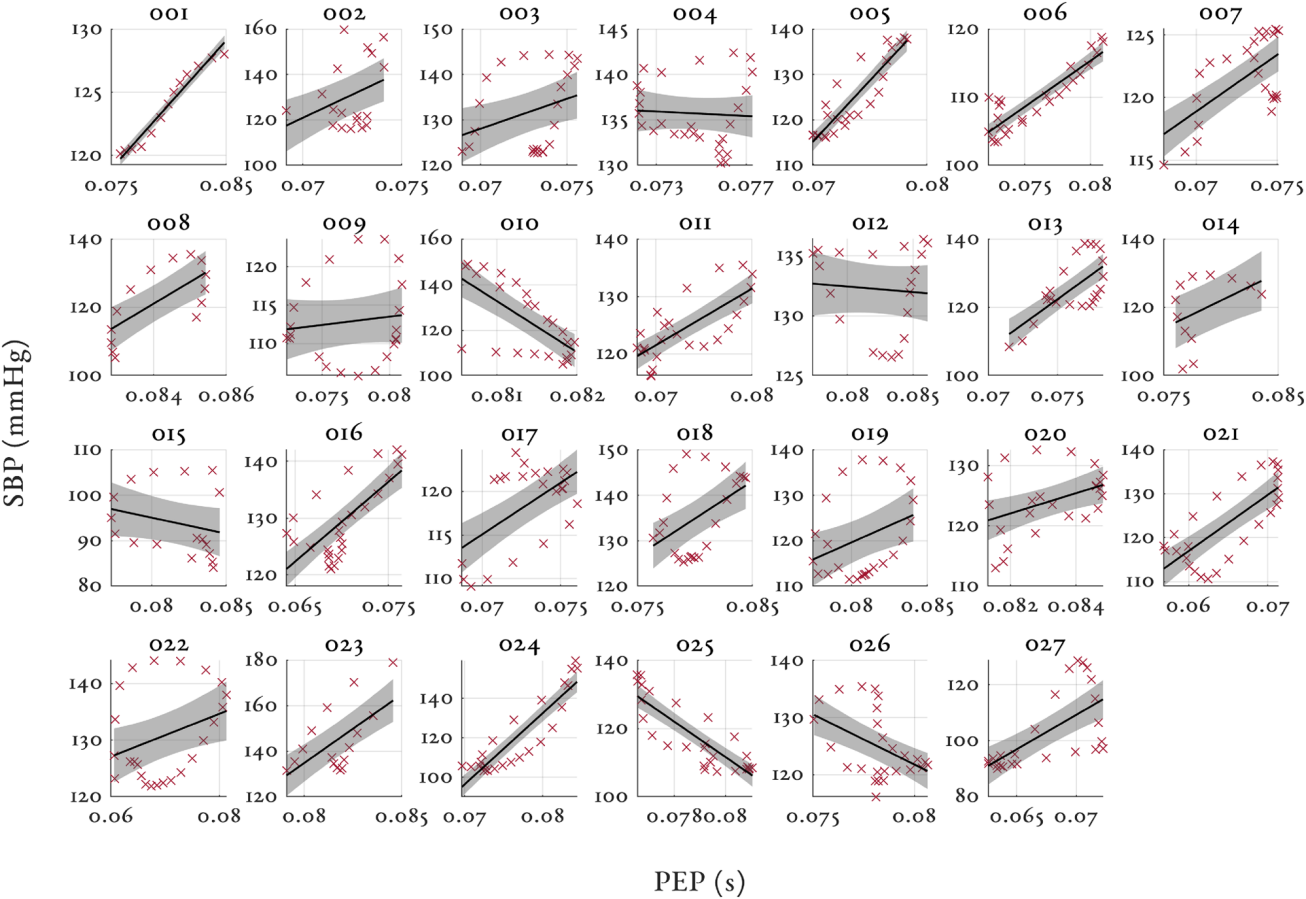
Individual relationships between SBP (mmHg) and PEP (s) for all volunteers in the study. The 95% confidence intervals are shown by the shaded region for each volunteer. Please note again the wide variation in y-axis scaling.

**Figure 5:**
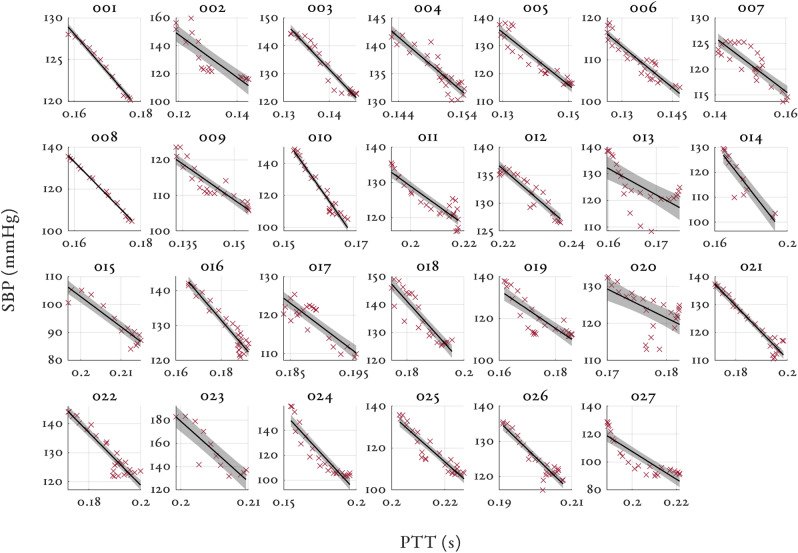
Individual relationships between SBP (mmHg) and PTT (s) for all volunteers in the study. The 95% confidence intervals are shown by the shaded region for each volunteer. Please note again the wide variation in y-axis scaling.

Figures 3, 4 and 5 shows the relationship between SBP and PAT, PEP and PTT respectively for all the volunteers in the study. The 95% confidence intervals are shown by the shaded region for each volunteer with the individual linear regression line. Similar figures for MAP and DBP can be found in Supplementary material B. Table 2 shows the median and interquartile range (IQR) correlation coefficients for PAT, PTT and PEP to SBP, MAP and DBP for all the volunteers in the study. Both PAT and PTT had strong negative correlations ($r < -\,0.8$) with BP. PTT had marginally larger correlation coefficients and tighter interquartile range (IQR) than PAT. PEP showed a moderately strong positive correlation ($r > 0.62$) with BP and had a large IQR.

**Table 2:** Correlation coefficients for PAT, PTT and PEP to SBP, MAP and DBP for all the volunteers in the study.

	PAT	PTT	PEP
SBP	− 0.89 (0.10)	− 0.92 (0.09)	0.42 (0.53)
MAP	− 0.86 (0.16)	− 0.91 (0.12)	0.51 (0.56)
DBP	− 0.84 (0.30)	− 0.85 (0.21)	0.61 (0.64)

Values are given as median (IQR).

### Impact of PEP

**Figure 6:**
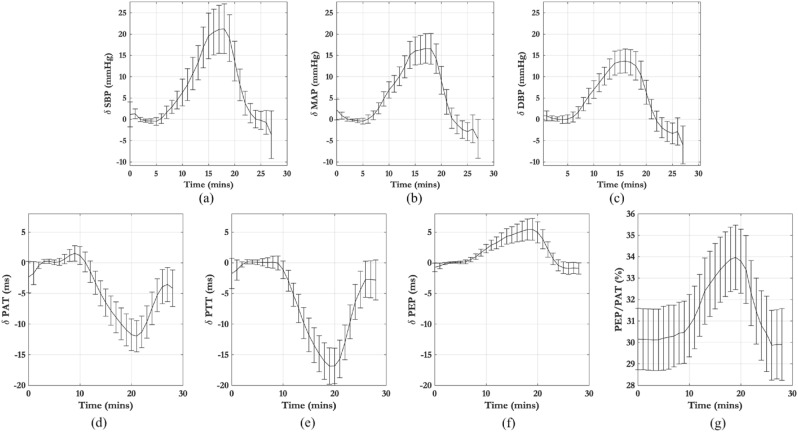
Average deviations from baseline for all participants in the study for (**a**) SBP, (**b**) MAP, (**c**) DBP, (**d**) PAT, (**e**) PTT and (**f**) PEP. (**g**) variations in PEP as a percentage of PAT from the start to the end of the study. The error bars show the 95% confidence intervals.

Figure 6 shows the average deviations ($\delta$) in SBP, DBP, MAP, PAT, PTT and PEP from their baseline values for all volunteers in the study. The baseline values were computed as the median value during the 5-min resting period at the start of the study. The error bars show the 95% confidence intervals. On average SBP, MAP and DBP increased by 21.1 mmHg ($\pm \,13.74$ mmHg), 17.2 mmHg ($\pm \, 8.28$ mmHg) and 13.8 mmHg ($\pm \, 7.03$ mmHg) respectively from their baseline values. PAT and PTT decreased by 11.96 ms ($\pm \, 6.57$ ms) and 16.84 ms ($\pm \,7.50$ ms) respectively. PEP increased by 5.46 ms (± 4.51 ms). Figure 6d shows the change in PEP as a proportion of PAT across the study, with a mean value of 30.2% at the start of the session, rising to 33.97% at the time of peak infusion.

### Models for estimating BP from PAT or PTT

Table 3 shows the RMSE, Mean Absolute Error (MAE) and Mean Absolute Deviation (MAD) values between the estimated BP and the reference BP when computed using the 6 models reviewed. As Poon et al.^16^ did not define a model for MAP, only SBP and DBP were computed for all models. All a posteriori models significantly outperformed the population-based models. Generally, DBP had a lower estimation error in comparison to SBP. For all models, apart from Gesche and Fung, PTT was a better predictor of BP than PAT. The best performing model was the inverse squared model.

**Table 3:** Evaluation of different models for estimating BP from PAT or PTT.

	A posteriori models					Population-based models				
	Model		RMSE	MAE	MAD	Model		RMSE	MAE	MAD
PAT	BP = $\frac{{\text{a}}}{{\text{PAT}}}$ + b	SBP	5.50	3.63	4.14	Poon^16^	SBP	11.04	7.10	8.46
		DBP	4.49	3.42	2.92		DBP	8.37	6.12	5.71
	**BP** = $\frac{{\mathbf{a}}}{{\mathbf{PAT}}^2}$ + **b**	**SBP**	**5.48**	**3.61**	**4.12**	Gesche^18^	SBP	43.76	21.63	38.06
		**DBP**	**4.49**	**3.42**	**2.92**		DBP	46.70	22.93	40.71
	BP = a$\times$ln(PAT) + b	SBP	5.53	3.66	4.15	Fung^37^	SBP	8.51	5.70	6.33
		DBP	4.50	3.42	2.92		DBP	8.02	5.90	5.43
PTT	BP = $\frac{{\text{a}}}{{\text{PTT}}}$ + b	SBP	3.98	2.82	2.81	Poon^16^	SBP	8.49	5.40	6.56
		DBP	4.02	3.12	2.54		DBP	7.72	5.64	5.26
	**BP** = $\frac{{\mathbf{a}}}{{\mathbf{PTT}}^2}$ + **b**	**SBP**	**3.91**	**2.78**	**2.76**	Gesche^18^	SBP	4542	1773	4185
		**DBP**	**4.01**	**3.12**	**2.53**		DBP	4546	1776	4188
	BP = a$\times$ln(PTT) + b	SBP	4.05	2.86	2.87	Fung^37^	SBP	30.69	20.77	22.61
		DBP	4.03	3.12	2.55		DBP	33.80	22.45	25.29

All values are given in units of mmHg. Best performing models for PAT and PTT are highlighted in bold.

**Figure 7:**
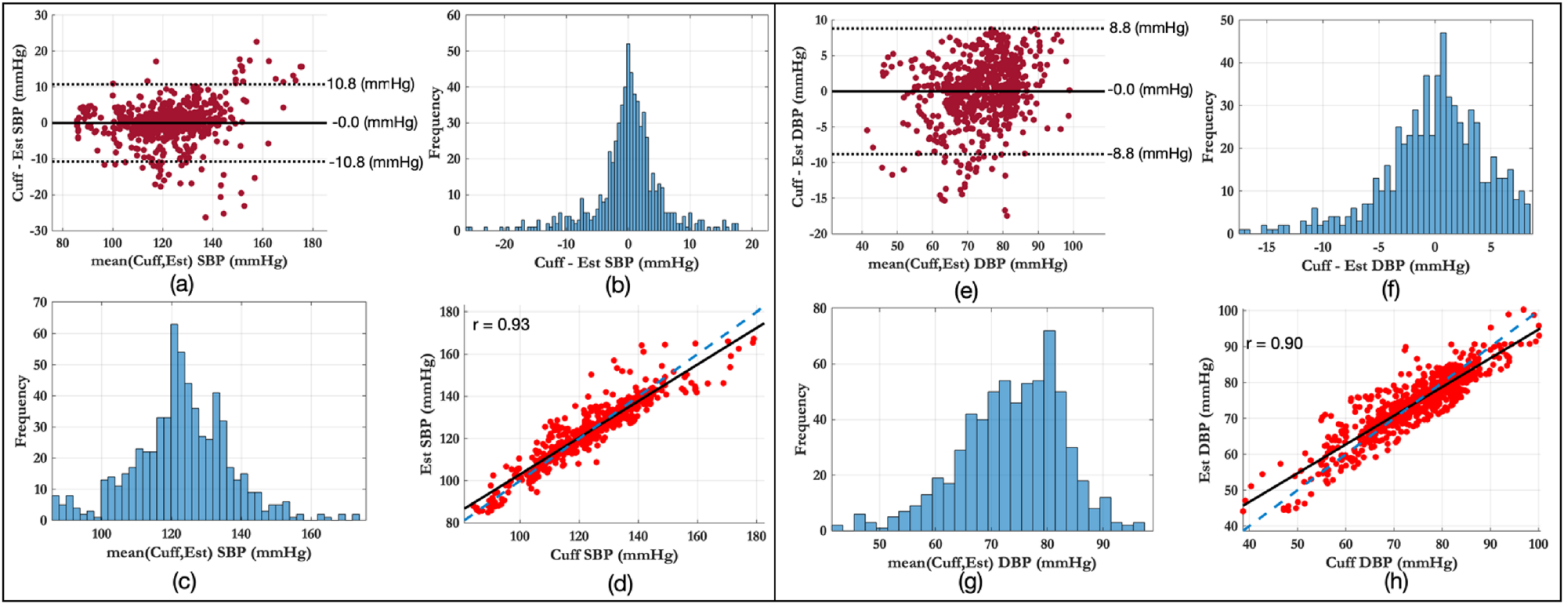
Agreement between the reference BP values from the sphygmomanometer cuff and the estimated BP values using PAT estimates and the inverse squared model. The left panel shows the results for SBP estimation, the right panel shows the results for DBP estimation. (**a**,**e**) The Bland–Altman plots shows no underlying bias. (**b**,**f**) The differences between the reference and estimated BP values. (**c**,**g**) The distribution of the mean values show that the BP values are within the expected physiological range. (**d**,**h**) The correlation is high, with an r of 0.93 for SBP and 0.92 for DBP.

Figure 7 shows, in more detail, the agreement between the reference BP values from the sphygmomanometer cuff and the estimated BP using PAT and the inverse squared model across all volunteers in the dataset. The left panel shows the results for SBP, the right panel shows the results for DBP. Figure 7a and e present the Bland–Altman analysis, which shows that there was no underlying bias between the two measurements, although SBP had wider confidence intervals than DBP. The Bland–Altman analysis also highlighted that large values of SBP tend to have larger estimation errors. Figure 7b and f show the histogram of differences between the reference and estimated BP values. Figure 7c and g show the histogram of mean values which are within the physiological range expected. Figure 7d and h show the correlation coefficients, for SBP the correlation coefficient was 0.93, whereas for DBP it was 0.92.

## Discussion

This work describes the methods for the non-invasive estimation of BP in a healthy volunteer study in which BP changes were induced by the infusion of phenylephrine. Phenylephrine is an alpha-1 adrenergic receptor agonist which causes venous and arterial vasoconstriction, increasing cardiac preload (initial stretching of cardiac muscles) and afterload (pressure against which the heart has to work to pump blood around the body)^35^. The dosing regimen was guided by the pharmacist in the Medical Sciences Division Ethics Committee (University of Oxford) who balanced the desired clinical effect against safety concerns surrounding bradycardia and a potentially rapid increase in blood pressure. Among our participants, we observed heart rate of around 40 beats per minute at peak infusion using this regimen. These decisions are outlined in the study protocol^38^. Our aim was to induce vasoconstriction using weight-based dosing protocol, rather than be BP-target driven. On average, we achieved an increase of approximately 20 mmHg in systolic blood pressure, with maximum increase of 40 mmHg in a subset of participants. One of the key advantages of this study was that, in a relatively short period of time, and without causing large movement, the volunteers experienced a wide range of BP values (see Fig. 6). This helped to validate algorithms for non-invasive estimation of BP.

### Relationship between BP and PAT or PTT in our dataset

Figure 2 shows the typical vital-sign response to the phenylephrine infusion for a 28-year-old female. As the dose increased, heart rate (HR), PAT and PTT decreased, whereas BP and PEP typically increased. $SpO_{2}$ and RR had no significant correlations to the dose of phenylephrine. In this work, the data from the BP cuff was processed using a cubic smoothing spline. To the authors’ knowledge, the only method for processing the BP cuff time series that has been used in the literature is a simple low-pass filter^29^. However, this assumes an evenly-sampled time series and requires a defined frequency range of interest.

PAT is inversely correlated with BP and the majority of volunteers had their own unique and strong relationship between changes in PAT and BP (see Fig. 3). As shown in Table 2, the absolute correlation between PAT and BP was high, with the correlation coefficients consistently higher than the values reported in the literature (see Mukkamala et al.^9^). This suggests that the complex pathways that link changes in BP to changes in PAT are likely to be associated with the pathways affected by phenylephrine. The correlation coefficients between PAT and BP were relatively consistent for SBP, MAP and DBP whereas in the literature, PAT is shown to have a much stronger relationship with either DBP^21,39^ or SBP^40,41^. All individuals had their own unique gradient defining the relationship between BP and PAT. The gradients varied from − 2946 to − 470.64 mmHg/s in our dataset. The gradient of the PAT to BP relationship is thought to reflect arterial properties such as compliance and resistance^9^. Shallow gradients are thought to be associated with young, healthy individuals. Conversely, steeper gradients are thought to be associated with more elderly individuals^42^.

There are several *caveats* to interpreting the relationship between BP and PAT. At points along the arterial tree where there are significant changes in impedance (such as at points of arterial branching), the difference in impedance to flow results in back propagation of part of the pressure wave. Thus, at any point in the arterial tree, the pressure wave can be considered a summation of forwards and backwards travelling waves. As a result, the proximal and distal waveforms used to compute PAT do not differ by simply a time delay but also by shape and amplitude^9^. This is a significant source of errors in PAT estimation^43^. To tackle this problem, fiducial points close to the onset of the PPG are typically used as they are thought to be the least impacted by reflected waves^44^. In this work, the intersecting tangents method was used to define the fiducial point of the PPG^9^.

Due to the impact of phenylephrine, smooth muscle activation was the dominant component regulating changes in the arterial stiffness. As the distal measurement site was located in the peripheral arteries, this effect cannot be considered negligible. This might explain why such large variations in gradients were found in this study, due to individual responses to the medication. The precise clinical effect of the weight-based dosing of phenylephrine (see “Methods” section) in an individual would depend on the balance between their sensitivity to the increase in afterload, the effect of bradycardia on cardiac filling (therefore contractility, the Frank–Starling law^45^), and the proportion of venous/arterial action of phenylephrine causing the increase in preload and afterload.

Additionally, we hypothesise that, in some volunteers, the dominant smooth muscle component may have given rise to a hysteresis effect, where the response is different between the period of dose escalation to the period of washout. This effect has been highlighted previously during an exercise test^46^, where the level of hysteresis was proportional to higher levels of ANS activity and smooth muscle tone. As a result, there are different baroreflex responses when blood pressure is rising or falling. We quantified the effect of hysteresis as differences in the PAT or PTT calibration gradients during the periods of dose increase and washout. Due to the small sample size, the significance of the hysteresis effect was examined using a Wilcoxon rank sum test on the magnitudes of the dose increase and washout gradients. Despite being clearly evident in a number of individuals, there was no statistically significant difference in gradient values found across the whole cohort or in gender and age subgroups. Equally, there was no evidence of differences in hysteresis effects present between PAT and PTT, suggesting that PEP does not play a significant contributing factor to PAT hysteresis. It should be noted, however, that the presented study was a healthy volunteer study and an examination of hysteresis was not a primary objective. Therefore, more work should be performed to examine this effect. For example, the hysteresis effect during multiple perturbations of BP should be examined over a longer duration.

### Impact of PEP

It has also been suggested that changes in PAT can often be significantly driven by changes in PEP^14^, making PAT an unreliable surrogate measure of blood pressure. As Wong et al.^22^ found, high correlations between PAT and BP do not necessarily suggest lack of influence of PEP, as this relationship could be driven primarily through changes in PEP. We found that PEP had a moderately strong, positive correlation ($r > 0.62$) with SBP, MAP and DBP. Phenylephrine is known to increase both preload and afterload (by vasoconstriction) which have opposing effects on PEP magnitudes. The positive correlation found in our dataset suggests that the afterload effect played a more dominant role. However, the large interquartile range (IQR > 0.6) indicates that the relationship between BP and PEP was multifactorial. The gradients of SBP-PEP relationship varied from − 22,320 to 63,162 mmHg/s in our dataset. PTT had a marginal increase in median correlation coefficient and a marginal decrease in IQR compared to PAT (as shown in Table 2), suggesting that PEP was not a significant contribution to PAT in our dataset.

The maximum deviation from baseline of PEP in our study had a mean value of 5.5 ms with a standard deviation of 4.5 ms (see Fig. 6f). This is comparable to the range seen in Payne et al.^14^ of 7.6 (10.1) ms in volunteers under norepinephrine, which acts in a similar manner to phenylephrine. Payne et al.^14^, concluded that the changes in PEP were too significant to allow for BP estimation using PAT. In our study, while we experienced relatively similar magnitude of changes in PEP, both PAT and PTT had a very strong correlation with BP. This was because the changes in PTT, mean − 16.8 (± 6.1) ms, were considerably larger than the changes in PEP. Furthermore, Payne et al.^14^ claimed that PEP could could vary between 12 and 35% of PAT. In our study, we found that PEP contributed between 28.8 and 35.2% of PAT (see Fig. 6g), a much lower range of values. Therefore, while the changes in PEP cannot be neglected, its relative contribution to PAT was nearly constant and so changes in PTT accounted for most of the changes in PAT during the 30 min of the study.

However, it is important to note that there are reported experiments, such as changes in posture^24^ or exercise^47^, where PEP is a non-negligible component of PAT. These effects suggest that a simple constant coefficient fit on PAT/PTT, may not be the best approach. Using this dataset, we found promising results suggesting that adding terms for PEP as well as the interaction between PEP and PAT/PTT may help to normalise the model coefficients leading to a more generalised model. However, as this was deemed outside the scope of this paper it should be considered further in future work.

### Models for estimating BP from PAT or PTT

We implemented both a posteriori models and population-based models to estimate BP from PAT and PTT (see Table 3). A posteriori models assume a unique relationship between BP and PAT/PTT. The model parameters (slope and intercept) are estimated by least squares using all data points available for that individual (see Supplementary material C for an assessment on the number of data points required for accurate BP estimation using a posteriori models). All a posteriori models reported comparable RMSE, MAE and MAD values for SBP and DBP estimation via PAT or PTT. The inverse squared model reported slightly lower RMSE, MAE and MAD values, although the difference is small.

Population-based models approximate model parameters using averaged physiological constants (such as the density of blood), global averages and calibration. Three different population-based models were tested: Poon^16^, Gesche^18^ and Fung^37^, three of the most highly cited population-based models for BP estimation using PAT or PTT. In each case, the models were validated using PAT estimates rather than PTT estimates.

The Gesche model had the worst performance for both PAT and PTT. This model used population-based averages derived using PAT on a study with 13 volunteers. In our dataset, the model errors using PAT were too large (RMSE > 40 mmHg) for accurate BP estimation. In addition, its performance was significantly worsened when implemented using PTT estimates. The Fung model, which is derived from a model of laminar blood flow through a rigid pipe, performed well on PAT estimates. The model approximated the gradient of an inverse squared model based on the subject’s height. However, the results on BP estimation using PTT were significantly worse, with RMSE values greater than 30 mmHg.

Overall the Poon model performed the best of the population-based models. The Poon model is derived from a combination of the Moens–Korteweg equation^10^ and a model relating the elastic modulus of a vessel to the mean pressure of the fluid inside it^12^. Even though the model was initially validated on PAT estimates, its accuracy was improved when PTT estimates were used as the input instead. This is to be expected as PEP is not included in the modelling by Poon. However, even in this best case, the error values for the Poon model were still approximately twice the error values from the inverse square a posteriori model. The Poon model approximates a physiological parameter, $\gamma$, for all individuals. $\gamma$ relates the distending pressure in an artery to the elasticity of the arterial wall. This relationship varies from person to person and can change as individuals age. Shao et al.^48^ highlighted that the Poon model errors are particularly sensitive to variations in $\gamma$.

The relationship between changes in PAT or PTT and changes in BP is unique to each individual, and so population averages are not sufficient to describe the complex underlying relationship on an individual level. All three models do not account for the effect of smooth muscle contraction which in our intervention was the dominant factor affecting changes in elasticity. This may go some way to explain why these models performed so poorly in our dataset. We would therefore suggest that individualised profiles are needed for accurate tracking of BP.

#### Clinical implications

The findings presented in this paper have important clinical implications for ambulatory non-invasive monitoring of BP. Firstly, we present an experiment where the changes in PAT are dominated by changes in PTT as opposed to the PEP component, making PAT an accurate surrogate of BP. For effective ambulatory non-invasive monitoring of BP, the relationship between PEP and PTT needs to be further explored and documented so that modes of failure can be highlighted and addressed. Secondly, a posteriori models naturally outperform population-based models for BP estimation, but they are not without their limitations. In this study, estimation of the calibration curve required monitoring during a drug-induced increase in vascular tone. This set-up would not be viable to be developed for non-invasive blood pressure monitoring for the general population. Additionally, the calibration curve will likely depend on basal cardiovascular factors which cannot be assumed constant in ambulatory monitoring such as intravascular fluid volume, cardiac contractility, and caffeine intake. More work should investigate the long-term stability of the calibration parameters and their relationship to these basal factors^42,49^.

### Limitations

There are several limitations to the methodology used in this study that should be noted. PEP was estimated using the Lozano method^50^. This method avoids detecting the B-point (point of aortic valve opening) by detecting the C-point (maximum of ICG pulse) of the ICG and a derived relationship between the ECG R-peak and the ICG B and C points. However, van Lien et al.^51^ reported considerable errors between PEP estimation using the Lozano method and hand annotated PEP values, especially for extreme values. Other techniques for detecting the B-point exist^52,53^, however, in our study, the Lozano method produced considerably less noisy estimates than these techniques.

The ICG signal was sampled at 200 Hz, resulting in a time resolution of 5ṁs for the detection of the C-point. This could potentially lead to errors in the estimation of PEP. Supplementary material F provides a comparison of PEP estimates from the original 200 Hz signal against PEP estimates from an upsampled ICG waveform at 1 kHz using cubic splines. Although we found that this limitation did not significantly impact the results and conclusions of this paper, further studies should aim to record the ICG waveform at a high sampling rate, such as 1 kHz, especially to estimate a wide range of PEP values.

Measurements of BP using a sphygmomanometer cuff are susceptible to various forms of noise that can distort the readings. The oscillometric device used as a BP reference in this study was compliant with the IEC 60601-2-30/EN60601-2-30 and with the American National Standard for Electronic or Automated Sphygmomanometers (ANSI/AAMI SP 10/92)^54^ with a maximum mean error of ± 5 mmHg (± 0.7 kPa) and a maximum standard deviation of 8 mmHg (1.1 kPa). As these tolerances are of similar magnitude to the errors seen in this work, we suggest that some of the errors in our BP estimates are not only due to problems in the PAT or PTT time series, but also due to errors in the reference BP values.

Finally, our results were reported across a small number of homogenous healthy volunteers. Volunteers were screened to ensure that they did not have a history of cardiovascular disease or hypertension. Meaningful estimation of BP must be evaluated on large heterogenous datasets involving both free-living individuals and those diagnosed with cardiovascular disease.

### Conclusion

To conclude, PAT can be considered an approximation of PTT if the contribution of PEP is neglected. Previous studies^14^ have suggested that the contribution of PEP provides a significant limitation to BP estimation using PAT. We have shown that under an infusion of phenylephrine, changes in PTT were significantly larger than changes in PEP and so the use of PAT instead of PTT is justifiable. Blood pressure estimation using PAT is best used in settings for which changes in PTT are significantly larger than changes in PEP. One such area could be sleep, where typically a decrease in night-time BP is experienced^55^. Forouzanfar et al.^53^ reported similar changes in PEP during a sleep study compared to those we found in our study.

Finally, we have confirmed that population-based models do not adequately reflect the unique and individualised relationship between changes in BP and changes in PAT, and so the use of these models to estimate BP in a given individual is not justified.

## Methods

### Clinical study

30 healthy volunteers, with no history of cardiovascular disease, were recruited for the clinical study. The study protocol has been outlined previously^38^. The study took place at the Cardiovascular Clinical Research Facility within the John Radcliffe Hospital, Oxford, UK. This study was reviewed and approved by the Oxford University Research and Ethics Committee and Clinical Trials and Research Governance teams (R63796/RE001). All methods were performed in accordance with the relevant guidelines and regulations. Individual informed written patient consent was obtained from all the participants in the study to record the data and publish the results, including anonymised images.

**Figure 8:**
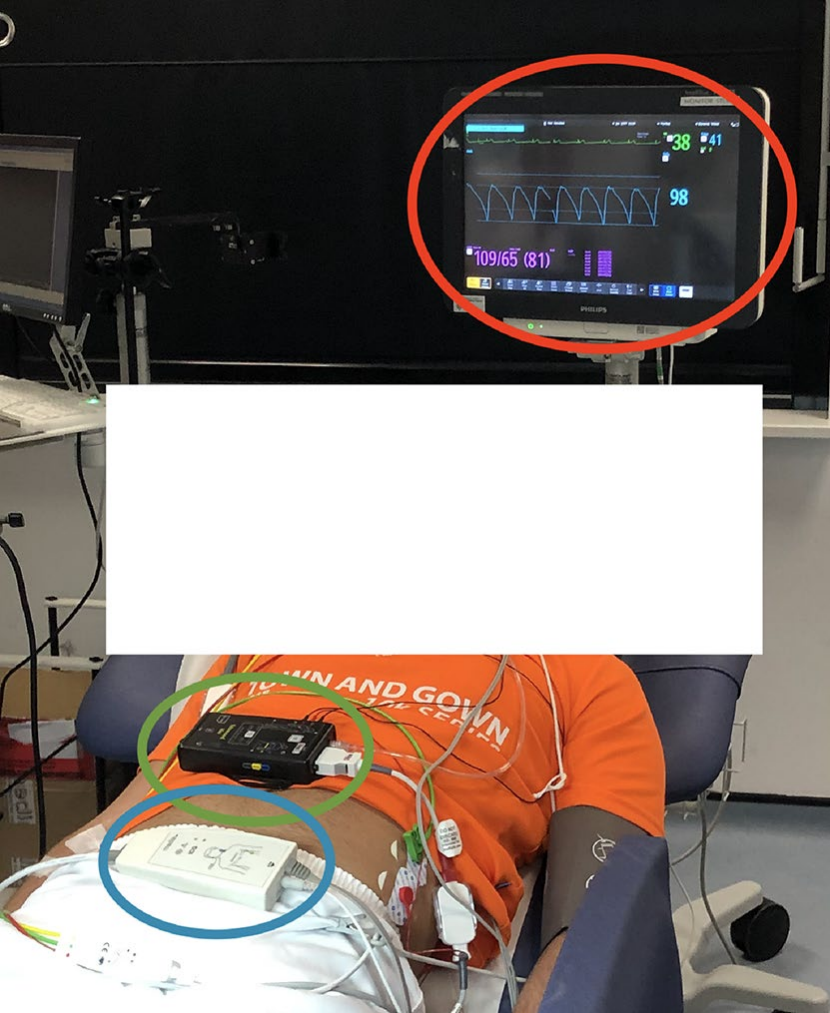
Equipment set up in our clinical study. The Philips patient monitor is circled in red, the Stowood device is circled in green and the CardioScreen device is circled in blue. Consent was obtained from the participant to use the image for publication.

Each session began with a 5-min resting period. Volunteers were then administered an infusion of phenylephrine, a vasoactive medication. Phenylephrine causes venous and arterial vasoconstriction and increases cardiac preload^35^. Phenylephrine was administered as an intravenous solution, starting at a rate of 0.2 mcg/kg/min with an increase of 0.2 mcg/kg/min every 1-min for 10 increments. Once the maximum rate was reached, the infusion and all monitoring remained constant for a further 6 min. Each session ended with an 8-min washout period. Volunteers were asked to refrain from ingesting caffeinated drinks an hour prior to the study visit as caffeine is a vasoconstrictor. Volunteers were positioned on their back with the head and trunk raised to between $15^{\circ }$ and $45^{\circ }$ (semi-Fowler’s position), see Fig. 8.

#### Instrumentation

Figure 8 shows the typical data collection set-up used in the study. Data was recorded from three sources: a Philips Intellivue MX800 patient monitor (Philips, Netherlands), circled in red; a Stowood Visi Black series polygraphy device (Stowood, UK), circled in green; and a CardioScreen 1000 non-invasive haemodynamic measurement and monitoring device (Medis, Germany), circled in blue. The three monitors were time-synced at the beginning of each session and the relevant software was operated from the same computer.

Reference vital signs (BP, heart rate) were recorded using the Philips patient monitor. A Philips comfort care M3001A BP cuff was wrapped around the upper left arm of the volunteer with the centre directly above the brachial artery as recommended^56^. The Philips patient monitor was installed with a Philips IntelliVue MMS measurement module. The ixTrend software (Ixellence GmbH, Germany) was used to record the data streams generated by the patient monitor.

The Stowood visi black series device is a portable device designed to record physiological signals for overnight studies. In our study, we used the Stowood device to record physiological signals (ECG, PPG and airflow) and reference vital signs (heart rate and $SpO_{2}$). The pulse oximeter (Masimo, USA) probe was placed on the middle finger of the volunteer’s right hand.

The CardioScreen device recorded the trans-thoracic bioimpedance signal (Z), ECG and HR. Four pairs of electrodes were placed on the patient’s neck and thorax. The outer electrodes injected an alternating current of 1.5 mA at a 86 kHz frequency. The inner electrodes measured the impedance changes occurring with each heartbeat. Table 4 gives a summary of the physiological parameters recorded by the Philips, Stowood and CardioScreen monitors.

**Table 4:** Physiological parameters and sampling rates recorded by the 3 devices used in this study.

Device	Name	Description	Sampling rate (Hz)
Philips	SBP, MAP, DBP	Blood pressure signal from the sphygmomanometer cuff	$\frac{1}{60}$
	HR	Heart rate	1
Stowood	ECG	1-Lead electrocardiography signal	256
	PPG	Photoplethysmography signal	512
	Airflow	Airflow from nasal cannula signal	32
	HR	Heart rate derived from Masimo pulse oximeter	5
	$SpO_{2}$	Oxygen saturation provided by Masimo pulse oximeter	5
CardioScreen	ECG	1-Lead electrocardiography signal	200
	Z	Thoracic impedance signal	200
	HR	Heart rate derived from the EGG signal	1

### Overview of the methodology

**Figure 9:**
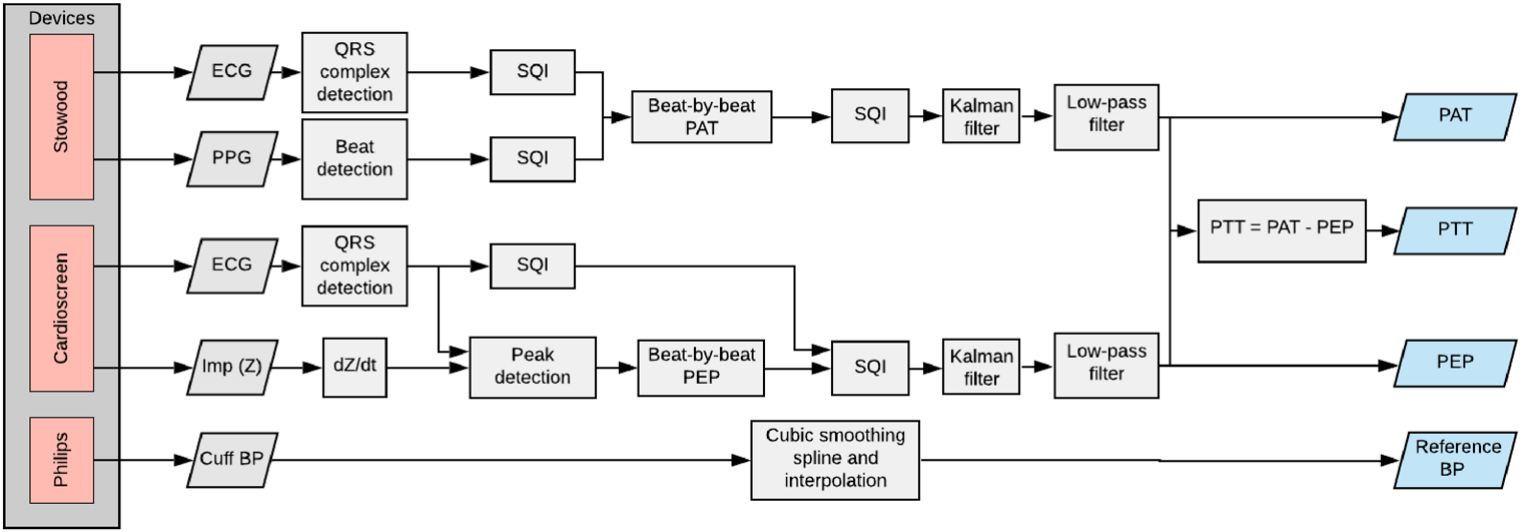
Overview of steps for estimating PAT, PEP and PTT and for processing the reference blood pressure signal from the blood pressure cuff values.

Figure 9 shows an overview of the steps taken for estimating PAT, PEP and PTT, as well as the steps for processing the reference blood pressure signal from thea cuff. Beat-by-beat PAT values were computed as the time delay from a proximal fiducial point on the ECG waveform to a distal fiducial point on the PPG signal recorded by the Stowood device. Periods of low quality from the ECG and PPG were detected by developing signal quality indices (SQI). The beat-by-beat PAT time series was processed by a Kalman filter to adjust the estimates based on the quality of the ECG and PPG waveforms. Subsequently, the baseline changes in PAT were extracted in order to be compared to the reference BP values. Beat-by-beat PEP was estimated using the ECG and ICG computed from the Cardioscreen device. PEP was estimated by tracking changes in the peak of the ICG signal and using a data-driven approach to relate this to changes in PEP. Poor quality periods of the PEP signal were detected by combining four signal quality indices (see below). Similarly, the beat-by-beat PEP time series was processed by a Kalman filter and baseline changes were extracted in order to be compared to reference BP values. PTT was computed as the difference between PAT and PEP. BP was recorded by the Philips monitor and was smoothed and interpolated to remove the effects of noise.

### Computing PAT

#### Detecting the proximal fiducial point from the electrocardiogram

The R-peak (point of left ventricular depolarisation) of the ECG waveform was used as the proximal site for PAT estimation. Therefore, accurate estimation of the QRS complex and an assessment of its signal quality was vital. To suppress the impact of baseline wander, the ECG was filtered using an 8th-order Butterworth infinite impulse response (IIR) high-pass filter with a cut-off frequency of 0.5 Hz. To suppress power-line interference, a 2nd order IIR notch filter with centre frequency at 50 Hz (the frequency of mains power in the UK) was used. The QRS complex of the ECG was detected following the work of Pan and Tompkins^57^.

#### ECG signal quality

An assessment of quality of the ECG waveform was required as the signal was affected by noise and motion artefacts. A signal quality index (SQI) is a beat-by-beat assessment of certain features of the ECG waveform. The SQI is a number between 0 and 1 (inclusive), representing a poor-quality or good-quality ECG beat respectively. The computation of the ECG SQI follows the work of Li et al.^58^. The overall SQI was computed by combining the following metrics: The agreement of R-peaks detected by two beat detectors; the kurtosis of the waveform; the instantaneous heart rate (IHR) computed using the interval between adjacent R-peaks; the analysis of the signal-to-noise ratio of the ECG signal. The acceptable limits for IHR was set as 30–90 bpm (see Fig. 1). All quality metrics were applied to a 10-s sliding window centred on each beat. The metrics were later combined to compute a single signal quality metric, SQI_ECG_, for each ECG beat. More details of the algorithm can be found in supplementary material D.

#### Detecting the distal fiducial point from the photoplethysmogram

The PPG waveform is a pulsatile signal indicating changes in blood volume. The PPG signal can be contaminated by many sources of noise. Most significantly, motion artefacts caused by movement or breathing alter the shape of the PPG waveform. A cascade of low and high pass FIR filters was used to band-pass filter the PPG signal around a valid heart rate range, taken to be between 30 and 90 bpm (see Fig. 1). The pre-processing filter was designed as a cascade of an 8th order low-pass IIR filter with a cut-off frequency at 1.9 Hz and a 60th-order high-pass FIR filter with a cut-off frequency at 0.2 Hz.

Villarroel^59^ designed a robust peak and onset detection algorithm for pulsatile signals, known as the Boxed Slope Sum Function (BSSF). The algorithm builds off the Slope Sum Function (SSF) from Zong et al.^60^ by adaptively tracking the upslopes of the signal. SSF enhances the slope of the pulse and suppresses the remainder of the waveform. Given $x_{k}$ as the filtered PPG signal at time *k*, the SSF was defined as:

1 $$\begin{aligned} \text{SSF}(i) = \sum _{k = i-w}^{i} u_{k}, \quad u_k = \quad {\left\{ \begin{array}{ll} \Delta x_k &{}\quad {\text{if}} \quad \Delta x_k > 0 \\ 0 &{}\quad {\text{if}} \quad \Delta x_k \le 0 \end{array}\right. } \end{aligned}$$

where *w* is the analysis window length and $\Delta x_k = x_k - x_{k-1}$. The window length *w* was chosen to be the typical duration of the upslope of the pulse, *w* = 170 ms. The BSSF time series was computed from the SSF signal by finding the zero-crossing points at the beginning of the upslope and at the end of the downslope for each pulse. Adaptive thresholding and a decision rule were applied to the upslope points in the BSSF signal (uBSSF) to identify the onset of each beat^59^. The peak was defined as the maximum value of the PPG signal between a pair of detected onsets. A 340 ms refractory period was applied to prevent multiple onset detections.

Wave reflections, due to impedance mismatch, interfere with the propagating wave. In this work, as phenylephrine significantly altered the vascular tone causing an increase in arterial stiffness, we found significant changes in the peak of the PPG (see supplementary material G). This made the peak of the pulse wave an unreliable marker for the arrival of the primary pressure wave. As a result, fiducial points located around the foot of the PPG pulse are commonly recommended for PAT estimation^9^. The intersecting tangents method was used to define the location of the foot of the PPG, as is done commonly in the literature^14,47,61^.

#### PPG signal quality

To assess the signal quality of the PPG, we followed the work of Villarroel^59^. Four different signal quality metrics were employed and combined together to provide a beat-by-beat SQI value. The metrics included: An assessment of clipping in the signal; sharp changes in amplitude; IHR outside of the physiological bounds; the deviation of beats from running-average window template. The range of acceptable limits for IHR was set as 30–90 bpm (see Fig. 1). All quality metrics were combined to compute a signal quality estimate, SQI_PPG_, for each distal fiducial point. More details of the algorithm can be found in supplementary material E.

#### Estimating beat-by-beat PAT

PAT was estimated by processing all proximal ECG R-peaks and locating distal PPG fiducial points prior to the following ECG R-peak. If there were none, or more than 1 PPG fiducial points located between two successive R-peaks, this indicated an error in the beat detection algorithm (for either the ECG or the PPG) and no PAT beat was computed. If there were exactly one PPG fiducial point, PAT was estimated as the time delay between the ECG fiducial point and the PPG fiducial point.

#### PAT signal quality

As PAT was computed from two independent waveforms, the likelihood of periods of low-quality signal due to (but not limited to) motion artefacts was high. However, a significant proportion of work described in the literature for estimating PAT do not document any attempt to reduce the impact of low-quality signals. We have only found two papers that perform an assessment of the signal quality of the ECG or PPG signals when estimating PAT, Escobar-Restrepo et al.^61^ and Zhang et al.^27^. In general, there is a need for a robust algorithm to reduce the impact of noise on the PAT time series. In this work, the SQI for each PAT beat was calculated by a combination of outlier detection from the PAT time series and the SQI metrics of the proximal ECG R-peak and distal PPG pulse for each PAT beat.

Outliers can result from noise, errors in the beat detection algorithms used, motion artefacts or other errors arising from the way the sensors were attached to the volunteer. Outliers were detected by constructing a moving-average window, in which each beat was given a score based on their deviation from the average of the window, computed as the median, med$_{w}$, of a sliding window of size 30 s with a step of 25 s. The deviation was computed as the median absolute deviation, MAD$_{w}$. These metrics were chosen as they are robust to outliers^62,63^. The outlier SQI was calculated for every beat *k* as:

2 $$\begin{aligned} {\text{SQI}}_{\mathrm{o}}(k) = \quad {\left\{ \begin{array}{ll} 0 &{}\quad {\text{if}} \;\, {\text{med}}_{w} - t \times {\text{MAD}}_w> {\text{PAT}}(k) > {\text{med}}_{w} + t \times {\text{MAD}}_{w} \\ 1 &{}\quad {\text{otherwise}} \end{array}\right. } \end{aligned}$$

*t* is a threshold set as 1.96 for outlier detection of the PAT time series. For normally distributed data, this provided a 95% confidence interval. The SQI of each PAT beat was subsequently calculated as:

3 $$\begin{aligned} {\text{SQI}}_{\mathrm{PAT}}(k) = {\text{SQI}}_{\mathrm{o}}(k) \times {\min }({\text{SQI}}_{\mathrm{ECG}}(k), {\text{SQI}}_{\mathrm{PPG}}(k) \end{aligned}$$

#### Processing PAT time series for comparison to BP cuff

In order to compare the beat-by-beat PAT time series with the reference BP values from the BP cuff we propose a three stage process: Kalman filter to adjust values based on their signal quality; interpolation to an evenly sampled time series; and baseline filtering in order to be compared to the low frequency sampled BP values.

A Kalman filter was applied to adjust PAT values based on their signal quality as has been previously done by Zhang et al.^27^. The Kalman filter is an algorithm to compute an estimate of the current state, $x_{t}$, of a linear dynamic system as a weighted average of the system’s previous state estimate and the current measurement, $z_{t}$. This reduces the impact of transient changes from noise and motion artefacts. The Kalman filter is a recursive estimator consisting of two stages: the prediction stage and the update stage. Full details of the Kalman filter algorithm can be found in Ref.^64^. We implemented a simple Kalman filter for a one dimensional signal, similar to Li et al.^58^. The prediction and update equations were:

Prediction stage:

4 $$\begin{aligned} {\hat{x}}_{t}^{-}= & {} {\hat{x}}_{t-1} \end{aligned}$$

5 $$\begin{aligned} P_{t}^{-}= & {} P_{t-1} + Q \end{aligned}$$

Update stage:

6 $$\begin{aligned}&K_{t} = P_{t}^{-} (P_{t}^{-} + R )^{-1} \end{aligned}$$

7 $$\begin{aligned}&{\hat{x}}_= {\hat{x}}_{t}^{-} + K_{t} (z_{t} - {\hat{x}}_{t}^{-}) \end{aligned}$$

8 $$\begin{aligned}&P_{t} = (1-K_{t})P_{t}^{-} \end{aligned}$$

where $\hat{x}_{t}^{-}$ and $\hat{x}_{t-1}$ are the prior and posterior state estimates, $P_{t}^{-}$ and $P_{t}$ are the prior and posterior error variances, $K_{t}$ is the Kalman gain that minimises the posterior error variance, *Q* is the state noise variance, *R* is the measurement noise variance and *t* indicates the time step. *Q* was set to 1. *R* was modified using the equation:

9 $$\begin{aligned} R_{t} = R_{0} \times e^{\frac{1}{{\text{SQI}}_{\mathrm{PAT}}(k)^{2}}-1} \end{aligned}$$

where $R_{0}$ was set to 1.

**Figure 10:**
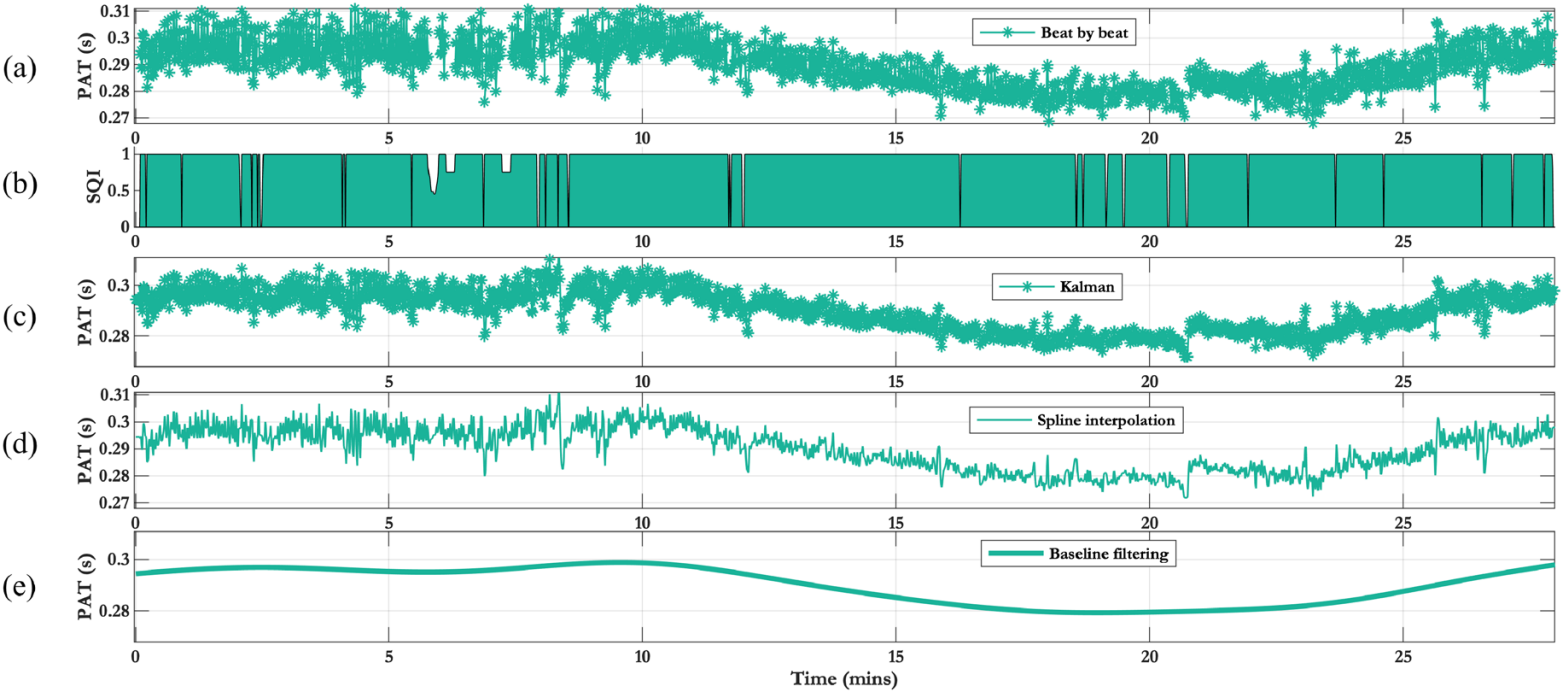
Overview of the PAT processing steps. (**a**) Beat by beat PAT time series, (**b**) SQI of the PAT time series, (**c**) Kalman filter output, (**d**) Spline interpolation of signal at 60 Hz, (**e**) Extracted baseline changes of PAT.

When comparing beat-by-beat PAT values to BP values from a sphygmomanometer cuff, the low sampling frequency of the cuff was insufficient to capture the high frequency changes in BP. Therefore, the PAT signal was filtered to capture changes in its baseline in order to be compared with the BP sampled at a much lower frequency. Any PAT beat with an SQI less than 0.8 was removed, as this was a cut-off deemed to indicate poor-quality beats. The irregularly sampled beat-by-beat PAT was then resampled at 60 Hz using cubic splines interpolation^65^. To capture changes in the baseline of PAT, a 6th-order low-pass filter with cut-off frequency of $1/(3 \times 60$) Hz was used, as suggested by Zheng et al.^29^. Figure 10 shows an overview of the PAT processing steps outlined above.

### Computing PEP

#### Estimating beat-by-beat PEP

The CardioScreen device recorded an ECG signal together with the trans-thoracic bioimpedance signal, Z. The ICG time series is the first-order time derivative of the bioimpedance signal, $\frac{dZ}{dt}$. As blood is a conductor, each heart beat produces a change in electrical impedance^66^. Therefore, the ICG signal has been shown to depend on haemodynamic parameters related to blood flow such as PEP^66^. As is commonly done in the literature^67,68^ to remove the influence of high frequency noise (such as mains interference) and low frequency drift (such as respiration), the ICG signal was band-pass filtered using a 4th order IIR filter with a cut-off frequencies set at 0.5 Hz and 40 Hz. The location of the R-peaks and beat-by-beat SQI of the ECG signal were computed using the steps outlined in previous sections on PAT estimation. The location of the R-peaks of the ECG defined the onsets of the beats of the ICG signal.

The C-point of the ICG signal is defined as the maximum of the ICG beat for each cardiac cycle^52^. In this work, and as is commonly done in the literature^52,69,70^, we estimated PEP using the Lozano method^50^. This method used a data-driven polynomial relationship which estimates PEP using the time delay from the R-peak to the C-point. As discussed by Lozano et al.^50^, the ICG beat may contain multiple maxima, in which case selecting the one with the largest amplitude may lead to incorrect PEP estimation. We, therefore, defined the C-point as the maximum of a low-pass filtered ICG signal. A 4th-order IIR filter with a cut off frequency of 5 Hz was subsequently used. This had the effect of combining two adjacent maxima and resulted in a less noisy PEP time series.

#### PEP signal quality

As the ICG was particularly vulnerable to noise, an adequate SQI to determine periods of good quality signal was vital for PEP estimation. We computed the beat-by-beat SQI of the PEP time series using four metrics. The first quality metric was computed from the proximal ECG signal, SQI$_{\mathrm{ECG}}$, processed using the methods outlined in the previous section and in the Supplementary material. The R-peaks of the ECG signal corresponded to the onsets of each ICG beat. The SQI of the *k*th ECG beat provided an initial measure of quality for each ICG beat, ICG$_{k}$.

The second SQI metric was based on the activity index (Act) of the ICG beat proposed by Forouzanfar et al.^53^. Variations in the ICG signal can indicate sources of noise such as: movement artefacts and sensor disconnect. To measure the ICG signal variations over each cardiac beat, the activity index was computed as:

10 $$\begin{aligned} {\text{Act}}(k) = \sqrt{\frac{1}{N(k)} \sum _{j=1}^{N(k)}\left( \frac{ICG_{k}(j)}{C} - \mu (k)\right) ^{2}} \end{aligned}$$

where *N*(*k*) is the length of ICG$_{k}$, $\mu (k)$ is the mean of the *k*th beat computed as the standard deviation of the normalised ICG$_{k}$:

11 $$\begin{aligned} \mu (k) = \frac{1}{N(k)}\sum _{j=1}^{N(k)}\left( \frac{ICG_{k}(j)}{C}\right) \end{aligned}$$

where C was a normalisation constant defined as the median peak-nadirs across all ICG beats in the session:

12 $$\begin{aligned} C = \underset{k}{\mathrm{median}}\text{(max(ICG}_k)- \text{min(ICG}_k)) \end{aligned}$$

Forouzanfar et al.^53^ defined a range of acceptable activity index values. Therefore, SQI$_{\mathrm{Act}}(k)$ was set as:

13 $$\begin{aligned} {\text{SQI}}_{\mathrm{Act}}(k) = {\left\{ \begin{array}{ll} 0 &{}\quad {\text{if}} \;\, 0.1> {\text{Act}}(k) > 0.4 \\ 1 &{}\quad {\text{otherwise}} \end{array}\right. } \end{aligned}$$

The third quality metric was designed to remove beats with a significant deviation in morphology from a running window template. Sheikh et al.^68^ proposed an SQI based on the computation of a three-stage ensemble averaged beat, ${\text{SQI}}_{\mathrm{EA}}$. A running window of length 30 s and step 25 s was used to construct the three-stage ensemble average. At each stage, and for each window, beats were assigned an ${\text{SQI}}_{\mathrm{EA}}$ value of 0 if they did not meet the stage criteria. An ensemble average for each window was then computed at each stage by averaging all beats that did not have an ${\text{SQI}}_{\mathrm{EA}}$ value of 0, starting 0.15 s prior to the R-peak with a duration equal to the median RR-interval of the window.

In the first stage, beats with an abnormal amplitude were assigned an SQI value of 0. Given a vector $Z = {z_{1}, z_{2} \ldots z_{M}}$, where $z_{k}$ is the amplitude of the *k*th ICG beat, M is the number of beats in each window. Beats were labelled as outliers if:

14 $$\begin{aligned} |z_k - \mathrm{median}(Z)| > 5 \times MAD(Z) \end{aligned}$$

At the second stage, the cross-correlation and lag of each beat with the ensemble average was computed. The lag of a beat was defined as the time shift at which the cross-correlation is maximum. Any beat with $|{\text{lag}}| > 50$ ms, or correlation coefficient < 0.5 was assigned an ${\text{SQI}}_{\mathrm{EA}}$ value of 0. At the final stage, the cross-correlation and lag of each beat with the ensemble average was computed, any beat with a non-zero lag to the ensemble average was circularly shifted to have zero lag. Any circularly shifted beat that had a correlation coefficient < 0.8 was assigned an ${\text{SQI}}_{\mathrm{EA}}$ value of 0.

The last quality metric was computed by detecting outliers from the PEP time series. As performed by Forouzanfar et al.^53^, outliers of the PEP time series were located using the outlier detection method previously outlined for the SQI of the PAT time series (SQI_o_) with a 60-s window, 55-s window step and a deviation threshold value of 5. The outlier detection algorithm was repeated until no more outliers were detected. All detected outliers were assigned an SQI_o_(*k*) value of 0.

Once the four proposed SQI metrics were computed, the final beat-by-beat SQI of the PEP signal (SQI$_{\mathrm{PEP}}$) was derived as:

15 $$\begin{aligned} {\text{SQI}}_{\mathrm{PEP}}(k) = {\text{SQI}}_{\mathrm{ECG}}(k) \times {\text{SQI}}_{\mathrm{Act}}(k) \times {\text{SQI}}_{\mathrm{EA}}(k) \times {\text{SQI}}_{\mathrm{o}}(k) \end{aligned}$$

#### Processing PEP time series for comparison to BP cuff

Similar to the PAT time series, the PEP time series was processed by a Kalman filter using the SQI of the beats to weight the measurement noise. The PEP time series was then interpolated using a cubic spline at 60 Hz and filtered using a 6th order low-pass filter with cut-off frequency of 1/($3 \times 60$) Hz to extract changes in its baseline value for comparison to the low frequency sampled BP cuff.

### Computing PTT

PTT was computed as the difference between the PAT and PEP values during the times for which the two signals overlap and both time series are of good quality, defined as having an SQI greater than 0.8.

### Computing the reference BP values

Blood pressure was measured by a sphygmomanometer cuff attached to the left arm of the volunteer and recorded by the Philips monitor. Measurements of blood pressure using cuffs have known limitations depending on: posture, movement, cuff-inflation hypertension and cuff size^71,72^. The cuff was programmed to inflate every minute. However, errors in cuff inflation can result in the Philips device being unable to register an accurate estimate, resulting in a missed data point in the blood pressure time series. As all volunteers followed the same protocol, we expected that there was an underlying trend in blood pressure that was common for all volunteers. However, there might have been phase and amplitude differences between individuals.

The cuff data was processed using a cubic smoothing splines^73^ algorithm, allowing for both filtering of the noisy blood pressure time series and interpolation of the readings when the cuff data was missing, resulting in a sampling frequency of one sample a minute (1/60 Hz). Cubic smoothing spline defines a function, *f*(*t*), that minimises:

16 $$\begin{aligned} L = p \sum (y_i - f(t_i))^2 + \int f''(t)^2 dt \end{aligned}$$

where *t* is time, $y_i$ are the observed BP values from the cuff, occurring at time $t_i$, $f''(t)$ is the second derivative of *f*(*t*), *p* is a constant defined as $p_{\mathrm{SBP}}$, $p_{\mathrm{MAP}}$ and $p_{\mathrm{DBP}}$ independently for SBP, MAP and DBP respectively.

The first term in Eq. (16) minimises the error between the data from the cuff and the regressed signal at the observation times, $f(t_{i})$. The second term penalises the roughness of the regressed signal by minimising the integral of the second derivative of *f*(*t*). *p* defines the relative weight placed on the first term with respect to the second term. A very low value of *p* will result in the regressed function converging to a linear least squares estimate. A very high value of *p* will result in the smoothing spline converging to a cubic spline that passes through all data points. The resulting function, *f*, is a twice differentiable smooth function, which equates to a cubic spline with knots at $f(t_i)$.

**Figure 11:**
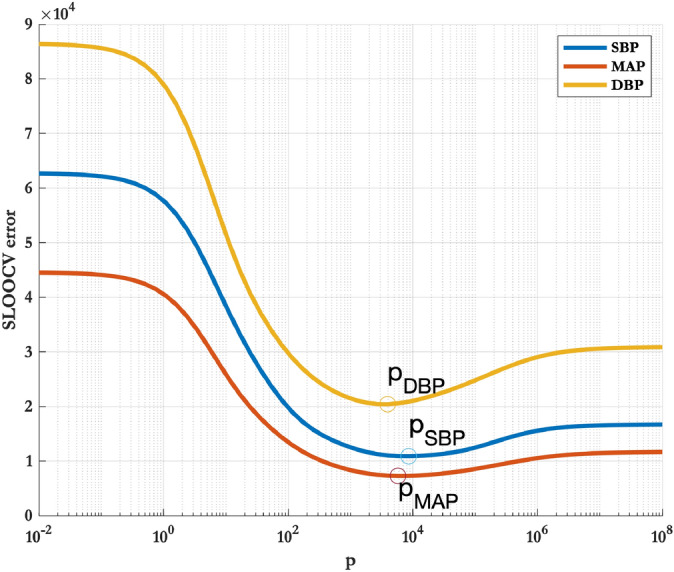
Determining $p_{\mathrm{SBP}}$, $p_{\mathrm{MAP}}$ and $p_{\mathrm{DBP}}$ by minimising SLOOCV across all volunteers in the dataset. The optimum values of $p_{\mathrm{SBP}}$, $p_{\mathrm{MAP}}$ and $p_{\mathrm{DBP}}$ are labelled.

As all volunteers were under the same protocol, we implemented $p_{\mathrm{SBP}}$, $p_{\mathrm{MAP}}$ and $p_{\mathrm{DBP}}$ values that were common for all of them. The error resulting from a *p* value for each volunteer was calculated by leave-one-out cross validation. The optimum *p* value for SBP, MAP or DBP ($p_{\mathrm{SBP}}$, $p_{\mathrm{MAP}}$ and $p_{\mathrm{DBP}}$ respectively) were therefore determined independently by minimising the summed leave-one-out cross validation error (SLOOCV) across all volunteers in the dataset. Figure 11 shows the values of SLOOCV against *p* values for SBP, DBP and MAP with the optimum values shown.

### Comparing PAT/PEP/PTT to blood pressure

The comparison between the PAT/PEP/PTT time series and the blood pressure measurement from the cuffs was a two-step process: time alignment and averaging. Various sources of delay between the PAT/PEP/PTT time series and the cuff data have been introduced, both through the instrumentation used and the processing steps outlined. These include errors in the time alignment of the Philips, Stowood and CardioScreen devices and timing errors in the cuff data. The signals were aligned to the blood pressure time series by subtracting the time delay computed as the lag at which the normalised cross correlation was largest. This process was done independently for PAT and PTT and for SBP, MAP and DBP, for each volunteer. The lag for PEP was assigned as the lag computed for PAT as this includes the PEP component. In order to compute the cross correlation, the two time series required to be sampled at the same sampling frequency. Therefore, for the purposes of time alignment only, the reference BP cuff data was interpolated using the cubic spline algorithm (outlined in the previous section), at the same frequency of the PAT/PEP/PTT time series (60 Hz).

The normalised cross-correlation, *R*, of two time series *x* and *y*, where *x* is delayed by $\uptau$ samples, is given by:

17 $$\begin{aligned} R_{xy}(\uptau ) = \frac{\sum \nolimits _{i-1}N(x_i - \mu _x)(y_{(i-\uptau )} - \mu _y)}{(N-1)\sigma _x \sigma _y} \end{aligned}$$

where $\mu _{x}$ and $\sigma _{x}$ are the mean and standard deviation of *x* respectively (and similarly for $\mu _{y}$ and $\sigma _{y}$). *N* is the number of overlapping data points. The time delay, $\tau$, was defined as:

18 $$\begin{aligned} \tau = \underset{\uptau }{\mathrm{argmax}}(-R_{xy}(\uptau ))) \end{aligned}$$

**Figure 12:**
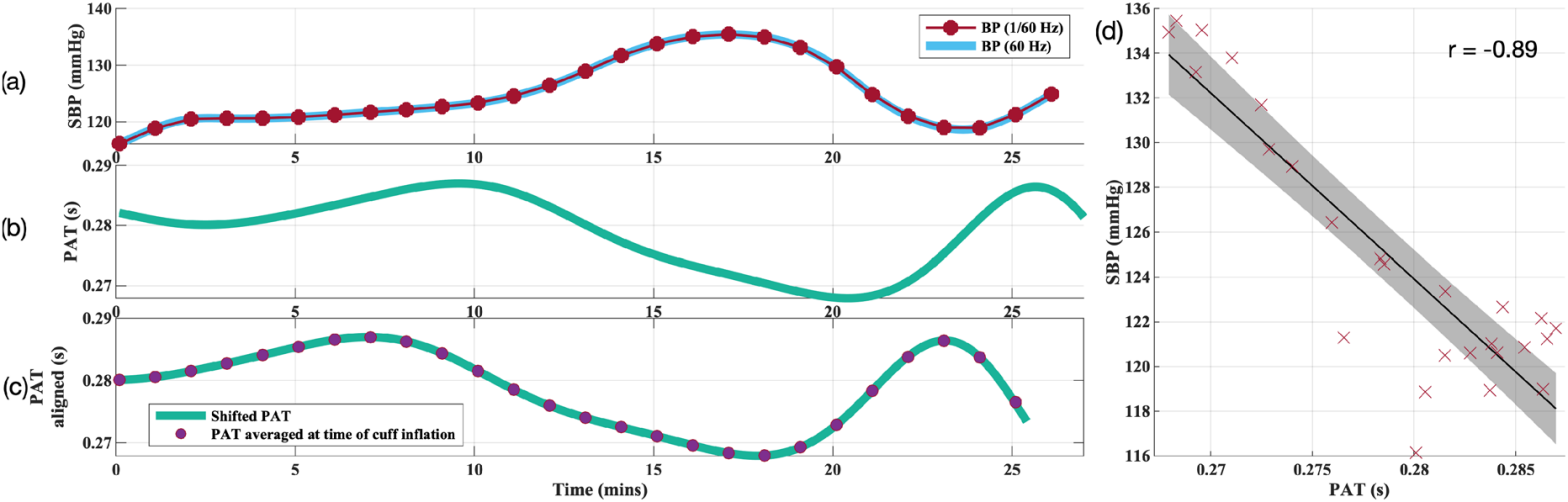
Example of the process for comparing the PAT time series to the SBP time series. (**a**) shows the SBP time series with the cuff inflations at once-a-minute and the 60 Hz interpolated signal used for signal alignment. (**b**) shows the processed PAT time series. (**c**) shows the aligned PAT time series shifted 148.5-s earlier and averaged PAT values at times of cuff inflation. (**d**) shows a scatter plot of the PAT and BP data points and the regression line through them.

Finally, the aligned PAT/PEP/PTT time series were averaged over 40-s windows, centred around each BP measurement. Figure 12 shows an example of the process for comparing the PAT time series to the SBP time series. Figure 12a shows the processed cuff data, sampled at both 1/60 Hz and 60 Hz. Figure 12b shows the PAT time series sampled at 60 Hz. Figure 12c shows the aligned PAT time series shifted 148.5-s earlier; the PAT signal averaged in a 40-s window centred on each BP datapoint is also shown. Figure 12d shows the BP data and the PAT data, plotted on the same plot.

We evaluated six different models proposed in the literature for the estimation of BP from PAT or PTT. The first 3 models use all datapoints available for that individual, and we refer to them as a posteriori models:

19 $$\begin{aligned}&\textbf{Inverse\, linear }^{21}: \qquad {\text{BP}}_{\mathrm{est}} = \frac{a}{\text{TT}} + b \end{aligned}$$

20 $$\begin{aligned}&\textbf{Inverse\,squared }^{74}: \qquad {\text{BP}}_{\mathrm{est}} = \frac{a}{\text{TT}^2} + b \end{aligned}$$

21 $$\begin{aligned}&\mathbf{Logarithm }^{43}: \qquad {\text{BP}}_{\mathrm{est}} = a\times \text{ln(TT)} + b \end{aligned}$$

where TT could be either PAT or PTT. The parameters *a* and *b* were approximated for each individual by least squares regression across all data-points available for that individual.

The remaining 3 proposed models allow for real-time estimation of BP using model parameters determined from population averages and physiological constants. We refer to these models as population-based models:

22 $$\begin{aligned} \textbf{Poon\,et\,al. }^{16}: \qquad {\text{DBP}}_{\mathrm{est}}&= \frac{\text{SBP}_{0}}{3} +2 \frac{{\text{DBP}}_{0}}{3} + \frac{2}{\gamma }{\ln }\left( \frac{TT_{0}}{TT}\right) - \frac{\text{SBP}_{0} - {\text{DBP}}_{0}}{3}\left( \frac{TT_{0}^2}{TT^{2}}\right) \end{aligned}$$

23 $$\begin{aligned} {\text{SBP}}_{\mathrm{est}}&= DBP_{\mathrm{est}} + ({\text{SBP}}_{0} - {\text{DBP}}_{0})\left( \frac{TT_{0}^2}{TT^{2}}\right) \end{aligned}$$

24 $$\begin{aligned} \textbf{Gesche\,et\,al. }^{18}: \qquad {\text{PWV}}&= \frac{0.5 \times {\text{height}}}{TT} \end{aligned}$$

25 $$\begin{aligned} {\text{BP}}_{\text{cal}}&= 700 \times {\text{PWV}}_{0}\times e^{-{\text{PWV}}_{0}} + 766{,}000 \times {\text{PWV}}_{0}^{9} \end{aligned}$$

26 $$\begin{aligned} {\text{BP}}_{\mathrm{est}}&= 700 \times {\text{PWV}}\times e^{-{\text{PWV}}} + 766{,}000 \times {\text{PWV}}^{9} - ({\text{BP}}_{\mathrm{cal}} - {\text{BP}}_{{0}}) \end{aligned}$$

27 $$\begin{aligned} \textbf{Fung\,et\,al. }^{37}: \qquad {\text{BP}}_{\mathrm{est}}&= \frac{(0.6\times {\text{height}})^{2} \times \frac{\rho }{1.4}}{TT^{2}} + \left( {\text{BP}}_{0} - \frac{(0.6\times {\text{height}})^{2} \times \frac{\rho }{1.4}}{{\text{TT}}_0^{2}}\right) \end{aligned}$$

where $\gamma$ = 0.031 mmHg$^{-1}$ and is a vascular parameter relating the distending pressure in an artery to the elasticity of the arterial wall^12^. $\rho = 1035$ kg/m$^{3}$ and is the average density of blood. All models require calibration of vital sign values at the start of the session. In this work, we defined the calibration period to include the first 2 cuff inflations, the average value of BP or TT during this period was taken as the calibration value and is labelled with a subscript $_{0}$.

The Poon model is derived from a combination of the Moens–Korteweg equation^10^ and a model relating the elastic modulus of a vessel to the mean pressure of the fluid inside it^12^. The Gesche model parameters were estimated by least squares regression on a group of 13 volunteers. The model was then evaluated on a group of 50 volunteers and reported a mean correlation coefficient between reference and estimated BP of 0.83. The Fung model was derived from a model of laminar blood flow through a rigid pipe.

The models were evaluated using root mean squared error (RMSE), mean absolute error (MAE) and mean absolute deviation (MAD):

28 $$\begin{aligned} {\text{RMSE}}= & {} \sqrt{\left( \frac{1}{n}\right) \sum _{i=1}^{n}\big (BP_{est_{i}} - BP_{cuff_{i}}\big )^{2}} \end{aligned}$$

29 $$\begin{aligned} {\text{MAE}}= & {} \left( \frac{1}{n}\right) \sum _{i=1}^{n}\left| BP_{est_{i}} - BP_{cuff_{i}} \right| \end{aligned}$$

30 $$\begin{aligned} {\text{MAD}}= & {} {\sqrt{\frac{1}{n}\sum _{i=1}^{n}\big (|BP_{est_{i}} - BP_{cuff_{i}}|-{\text{MAE}}\big )^{2}}} \end{aligned}$$

## Supplementary information

Supplementary Information.

## Data Availability

The datasets generated or analysed during the current study are not publicly available due to the sensitive and identifiable nature of our data, patient consent and restrictions of the ethics protocol to protect the privacy of patients involved in the study.
