# Supplementary material for: Pulse arrival time as a surrogate of blood pressure
Source: Sci Rep. 2021 Nov 23;11:22767. doi: 10.1038/s41598-021-01358-4 (PMC8611024; doi:10.1038/s41598-021-01358-4)
Supplement: Supplementary file 1 — Supplementary Information. [file 41598_2021_1358_MOESM1_ESM.pdf]

Pulse arrival time as a surrogate of blood pressure - Supplementary material

Eoin Finnegan[1,*], **Shaun Davidson**[1], **Mirae Harford**[1,2], **João Jorge**[1], **Peter Watkinson**[2], **Duncan Young**[2], **Lionel Tarassenko**[1], **and Mauricio Villarroel**[1]

[1]Institute of Biomedical Engineering, Department of Engineering Science, University of Oxford, UK
[2]Critical Care Research Group, Nuffield Department of Clinical Neurosciences, University of Oxford
[*]eoin.finnegan@eng.ox.ac.uk

Supplementary material A

Frequency spectrum of PAT

Figure 1 shows the frequency spectrum, computed by the fast Fourier transform (FFT), of the PAT signal for a typical volunteer. (a) shows the overall FFT which is dominated by a component close to DC. This component is thought to be driven by the slow changes in arterial stiffness due to the phenylephrine infusion. (b) shows the FFT zoomed in on the less dominant components. There are two frequency ranges of interest. The first is 0.083 Hz to 0.1 Hz, frequencies in this range are above the Nyquist limit of the cuff and so cannot be explained by the BP reference. The most likely explanation for power in this range may be the Traube-Hering-Mayer (THM) waves, often known as Mayer waves[1]. Mayer waves are oscillations in BP, with a frequency of around 0.1 Hz, thought to be due to oscillations of the sympathetic vasomotor tone of arterial blood vessels. The amplitude of the Mayer waves have been shown to be an indicator of sympathetic nervous activity[1]. The second component is 0.17 Hz to 0.35 Hz. This range is thought to be driven by respiration. Figure 2 shows the spectrogram of a PAT signal that has been high-pass filtered with a 0.1 Hz cut-off frequency. The reference RR from the nasal cannula is superimposed in red. Figure 2 shows that there is a clear respiratory component in the PAT signal which explains this second frequency range of interest.

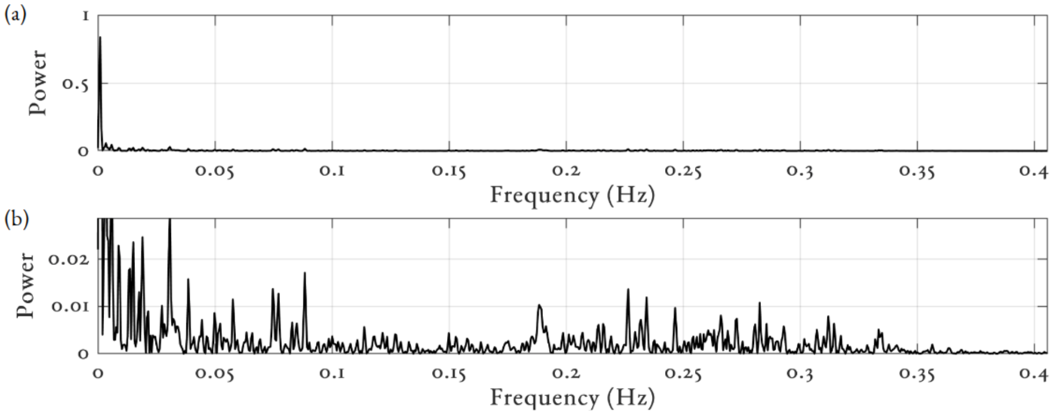

Figure 1. Frequency spectrum of PAT signal. (a) shows dominant component close to DC which is thought to be driven by changes in BP. (b) is zoomed in on less dominant components showing two frequency ranges of interest: (1) 0.083 - 0.1 Hz whose origin may be due to the Mayer waves[1], (2) 0.17 - 0.35 Hz which is thought to be driven by respiratory rate.

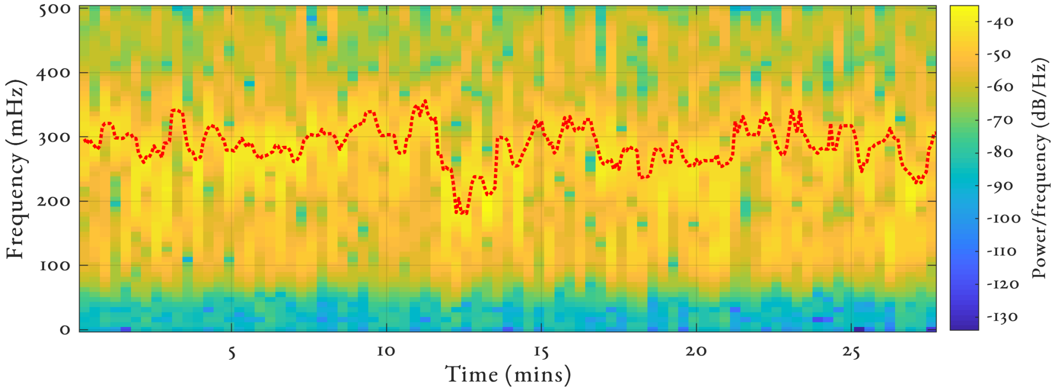

Figure 2. Spectrogram of a PAT signal that has been high-pass filtered with a 0.1 Hz cut-off frequency. The reference RR from the nasal cannula is superimposed in red.

Supplementary material B

MAP results

Figures 3, 4 and 5 shows the relationship between MAP and PAT, PEP and PTT respectively for all the volunteers in the study. The 95% confidence intervals are shown by the shaded region for each volunteer with the individual linear regression line.

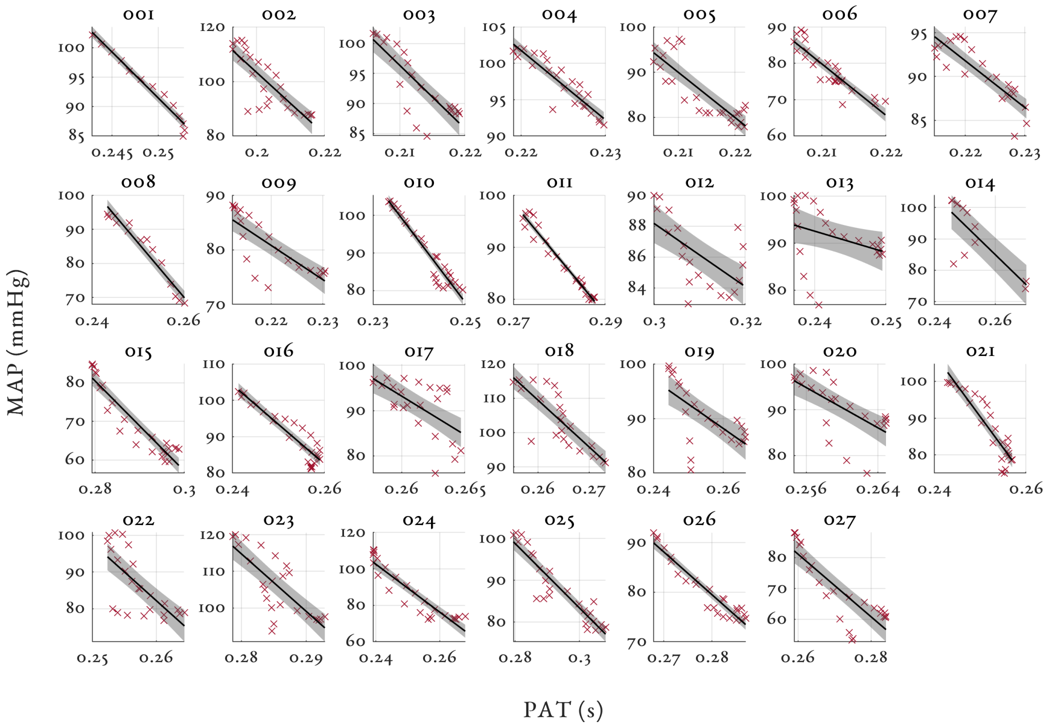

Figure 3. Individual relationships for between MAP (mmHg) and PAT (s) for all volunteers in the study. The 95% confidence intervals are shown by the shaded region for each volunteer. Please note again the wide variation in y-axis scaling.

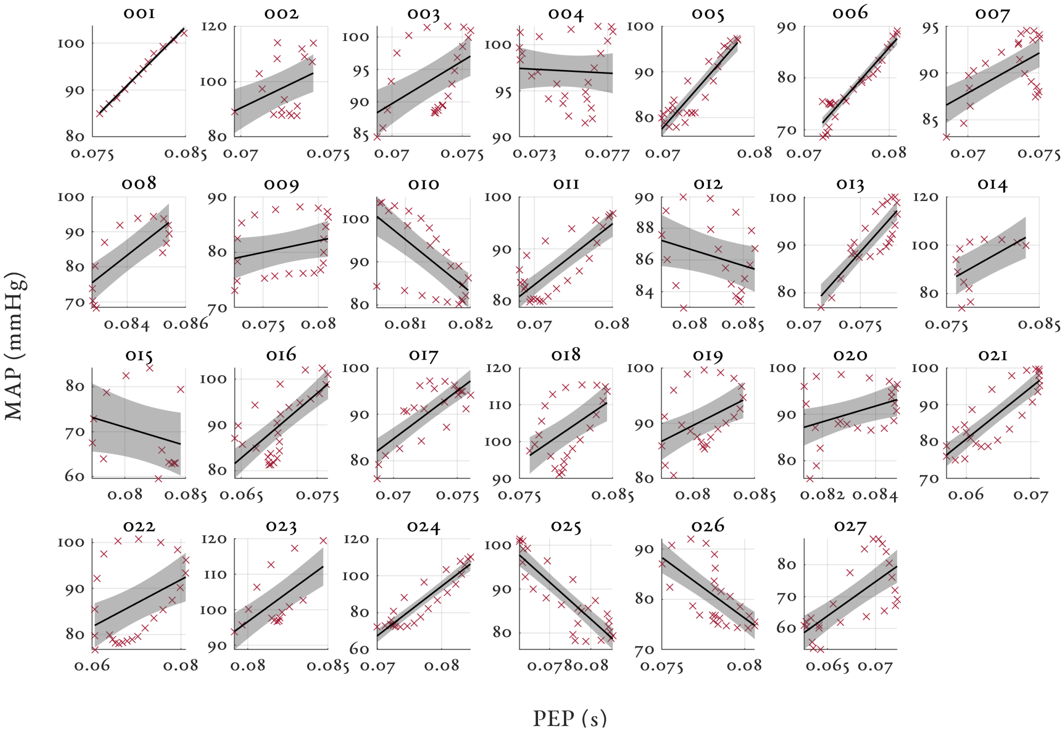

Figure 4. Individual relationships between MAP (mmHg) and PEP (s) for all volunteers in the study. The 95% confidence intervals are shown by the shaded region for each volunteer. Please note again the wide variation in y-axis scaling.

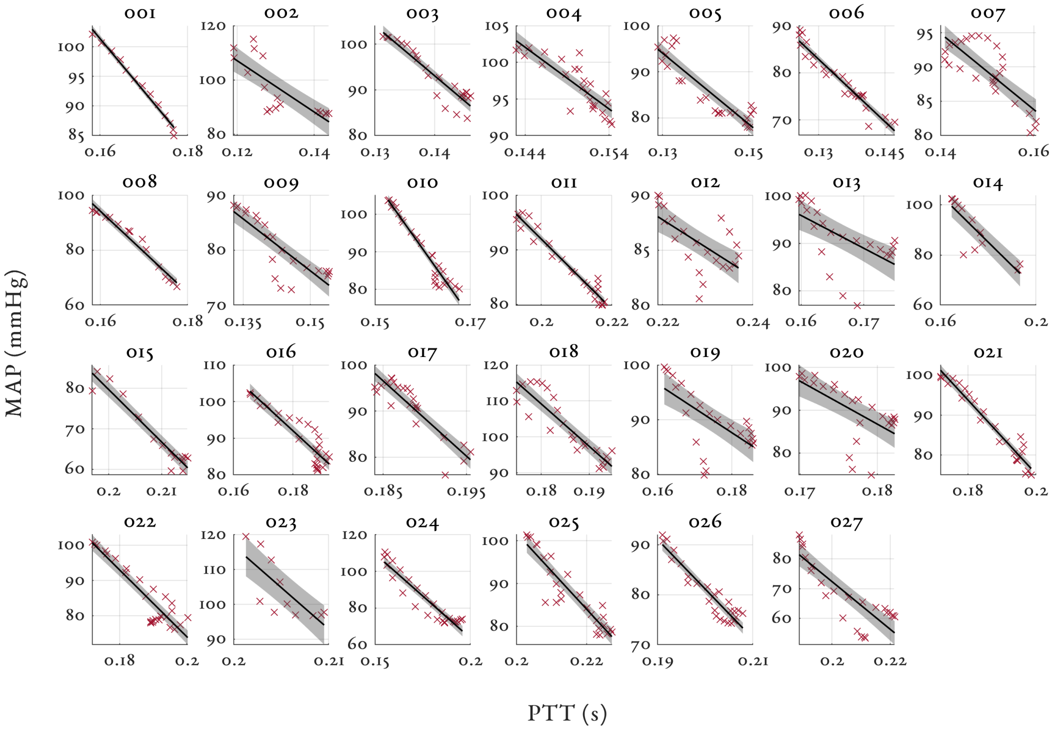

Figure 5. Individual relationships between MAP (mmHg) and PTT (s) for all volunteers in the study. The 95% confidence intervals are shown by the shaded region for each volunteer. Please note again the wide variation in y-axis scaling.

DBP results

Figures 6, 7 and 8 shows the relationship between MAP and PAT, PEP and PTT respectively for all the volunteers in the study. The 95% confidence intervals are shown by the shaded region for each volunteer with the individual linear regression line.

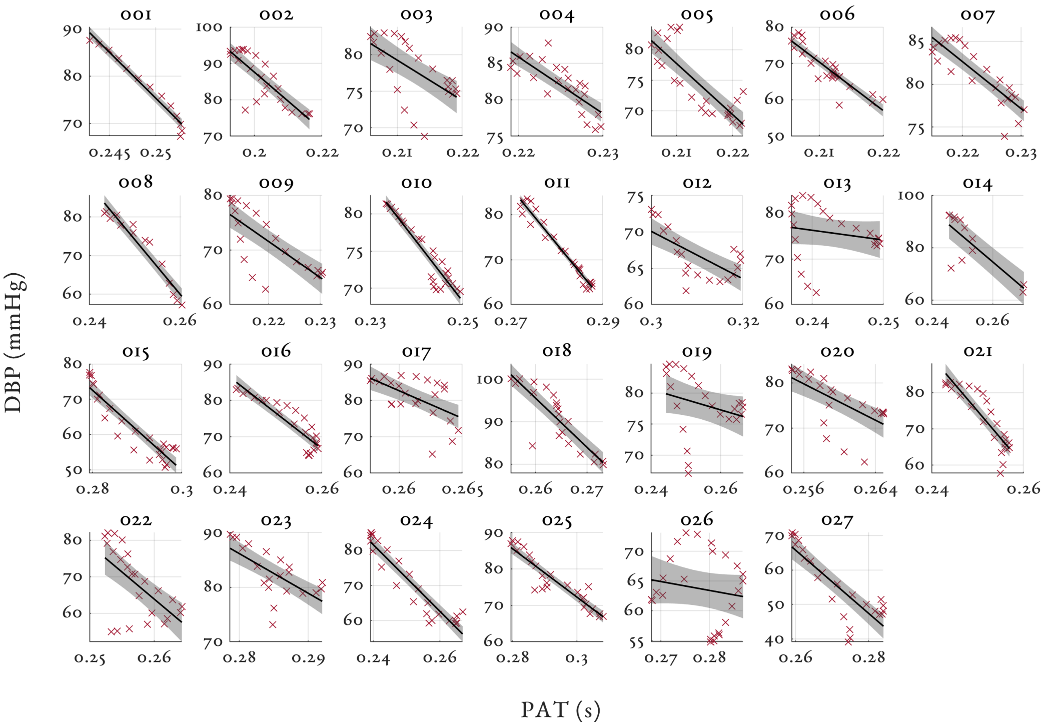

Figure 6. Individual relationships between DBP (mmHg) and PAT (s) for all volunteers in the study. The 95% confidence intervals are shown by the shaded region for each volunteer. Please note again the wide variation in y-axis scaling.

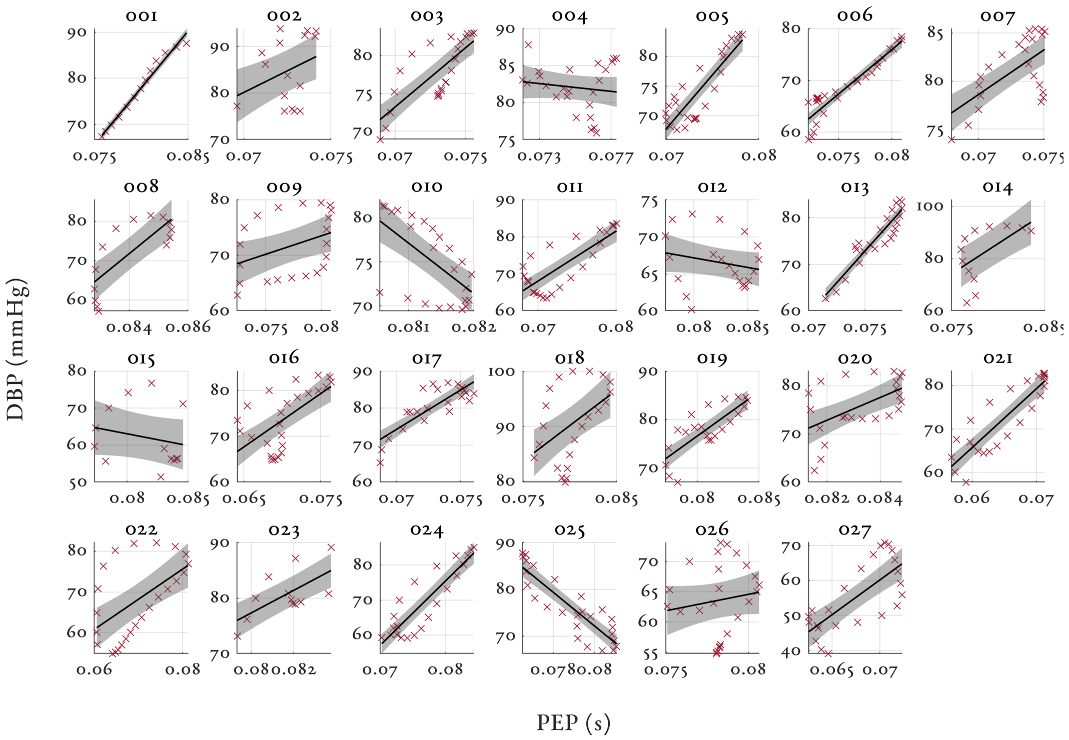

Figure 7. Individual relationships between DBP (mmHg) and PEP (s) for all volunteers in the study. The 95% confidence intervals are shown by the shaded region for each volunteer. Please note again the wide variation in y-axis scaling.

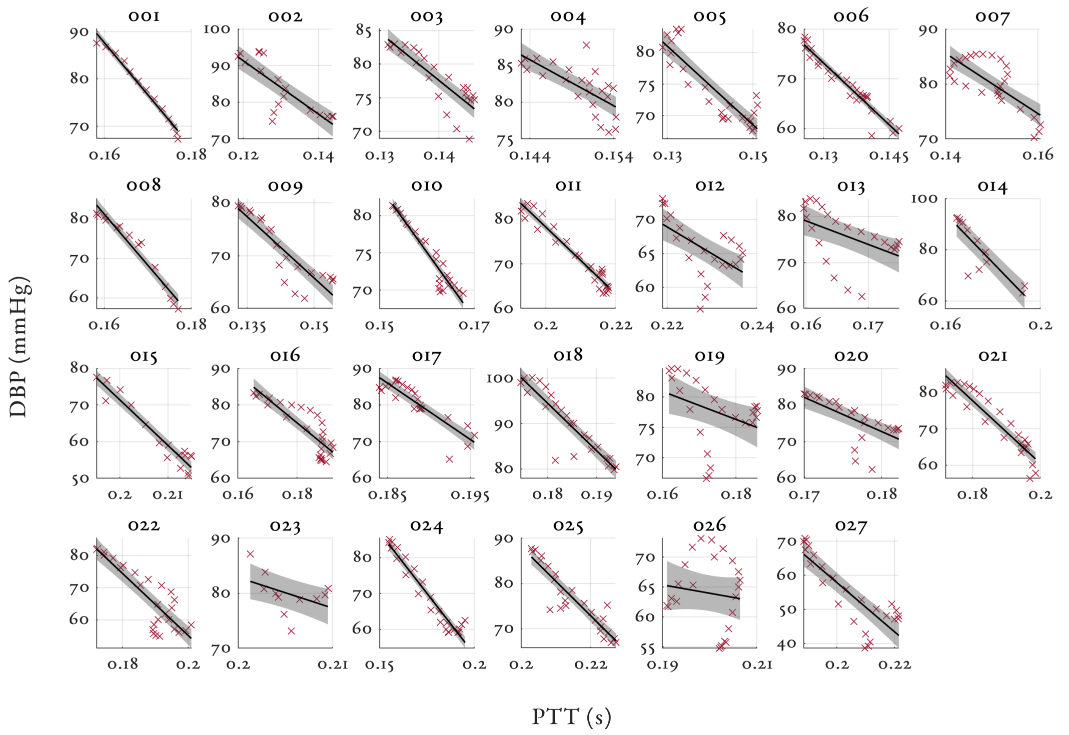

Figure 8. Individual relationships between DBP (mmHg) and PTT (s) for all volunteers in the study. The 95% confidence intervals are shown by the shaded region for each volunteer. Please note again the wide variation in y-axis scaling.

Supplementary material C

Number of cuff inflations required for accurate performance of *a posteriori* models

A posteriori model parameters (slope and intercept) could be estimated by two cuff inflations; however, more accurate estimates are made by using more cuff inflations. In this work we used all cuff inflations available for each individual to estimate the slope and intercept. Figures 9 and 10 shows improvements in the *a posteriori* inverse square model for estimating BP using PAT and PTT estimates respectively as more cuff inflations are used to estimate model parameters, starting from the first two cuff inflations. Dashed lines show the thresholds for standard requirements for blood pressure measuring devices set by Advancement of Medical Instrumentation (AAMI), European Society of Hypertension (ESH) and International Organisation for Standardisation (ISO)[2]. As can be seen, model performance plateaus once around 60 % of cuff inflations have are used. This can be approximated as around the point of maximum infusion demonstrating that to get an accurate assessment of an individuals calibration curve, a wide range of blood pressure values is required.

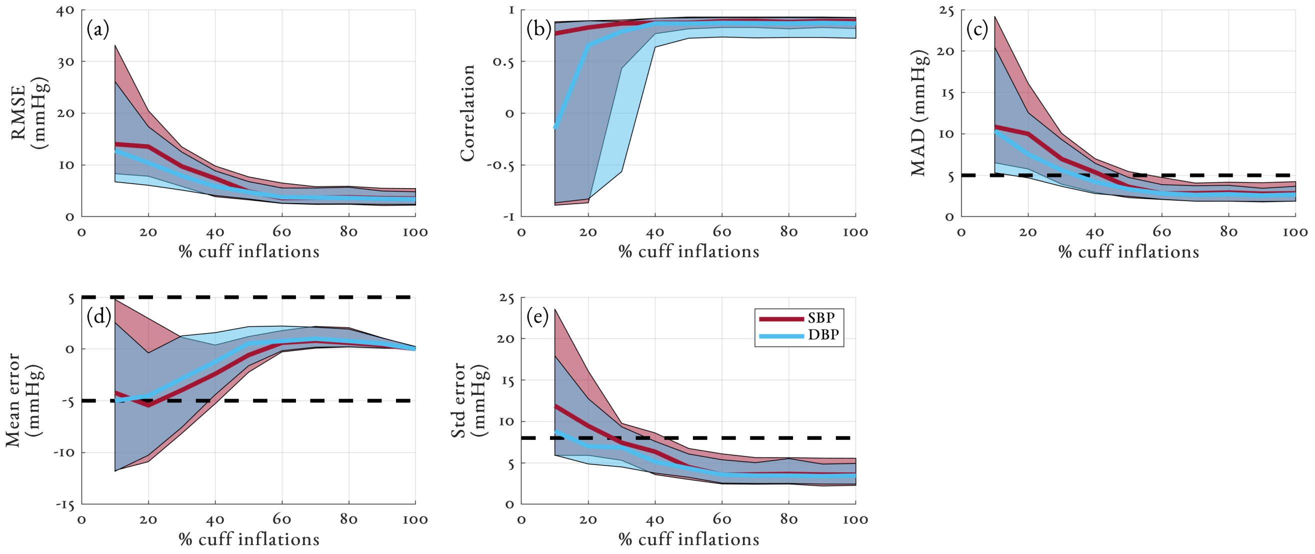

Figure 9. Improvements in performance metrics as more cuff inflations are used to compute the *a posteriori* model parameters (slope and intercept) using PAT estimates. Median values across all volunteers in the study are shown with interquartile ranges shown by the shaded region. Performance metrics are given as: (a) RMSE, (b) correlation coefficient, (c) MAE, (d) mean error, (e) standard deviation of error.

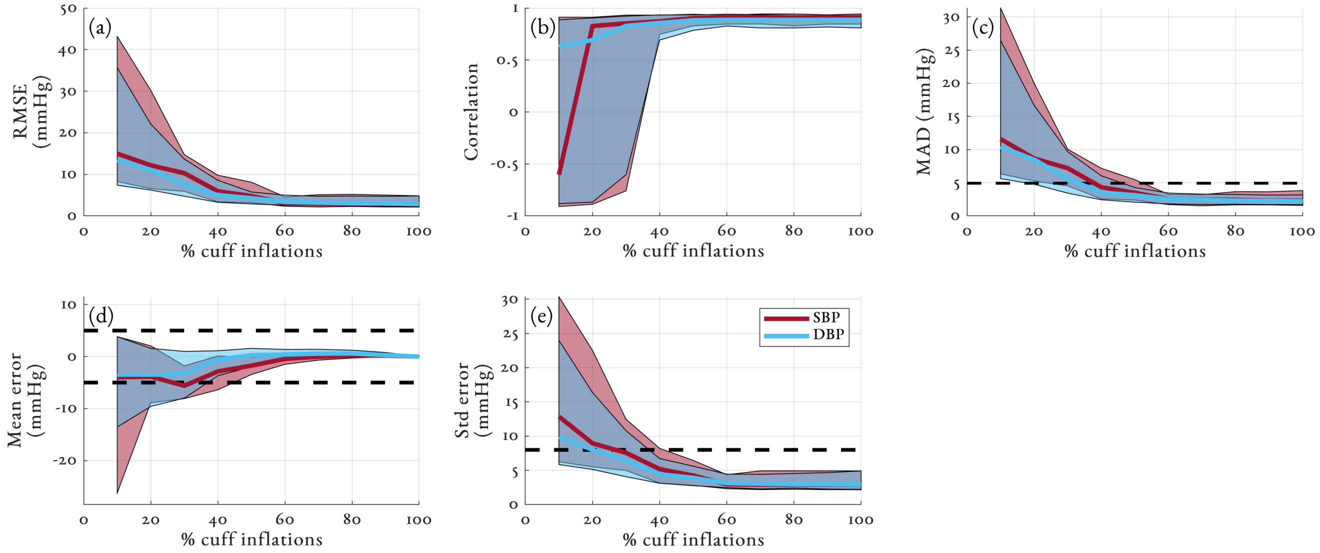

Figure 10. Improvements in performance metrics as more cuff inflations are used to compute the *a posteriori* model parameters (slope and intercept) using PTT estimates. Median values across all volunteers in the study are shown with interquartile ranges shown by the shaded region. Performance metrics are given as: (a) RMSE, (b) correlation coefficient, (c) MAE, (d) mean error, (e) standard deviation of error.

Supplementary material D

ECG SQI

For both PAT and PEP estimation, an assessment of the quality of the ECG waveform was required to remove periods where the ECG signal was affected by noise and motion artefacts. A signal quality index (SQI) is a beat-by-beat assessment of certain features of the ECG waveform. The SQI is a number between 0 and 1 (inclusive), representing a poor-quality or good-quality ECG beat respectively. The SQI of the ECG follows the work of Li et al[3]. The overall SQI was computed by combining the following metrics: The agreement of R-peaks detected between two beat detectors ($\mathrm{SQI_b}$) ; the kurtosis of the waveform ($\mathrm{SQI_k}$); the instantaneous heart rate (IHR) computed using the interval between adjacent R-peaks ($\mathrm{SQI_f}$); the analysis of the signal-to-noise ratio of the ECG signal ($\mathrm{SQI_{SNR}}$). All quality metrics were applied to a 10-second sliding window centred on each beat. The metrics were later combined to compute a single signal quality metric, $\mathrm{SQI_{ECG}}$, for each ECG beat.

Beat detection agreement (SQI_b)

bSQI employed two beat detectors: the Pan-Tompkins[4] and Wei Zong[5] QRS detector. The agreement between the two detectors was used to estimate the level of noise in the ECG signal. bSQI for the $\mathrm{k^{th}}$ beat centred around a window w was defined as:

$$\mathrm{SQI_b}(k) = \frac{N_{\mathrm{matched}(k,w)}}{N_{\mathrm{all(k,w)}}} \tag{1}$$

where N_{matched} is the number of beats for which both methods agree, within 150 ms as recommended by the Association for the Advancement of Medical Instrumentation (AAMI)[6], and N_{all} is the number of all beats that both algorithms detected minus N_{matched}.

Kurtosis (SQI_k)

From the central limit theorem, random uncorrelated processes, such as thermal noise, tend to have Gaussian distributions. Kurtosis measures the sharpness of the peak of a distribution and therefore provided a measure of how Gaussian the distribution is. The kurtosis of the ECG data x in a window w centred around the $\mathrm{k^{th}}$ beat was defined as:

$$\mathrm{K}(\hat{k}) = \frac{1}{N}\frac{\sum_{i=1}^{N}(x(i)-\mu)^4}{\sigma^4} \tag{2}$$

where N, μ and σ are the length, mean and standard deviation of the data x respectively. The kurtosis of a Gaussian-like distribution is approximately 3, and ECG contaminated with noise and artefacts usually has a kurtosis lower than 5[3]. Therefore, kSQI was defined as:

$$\mathrm{SQI_k}(k) = \begin{cases} 0 & \text{if} \quad \mathrm{K}(k) \leq 5 \\ 1 & \text{otherwise} \end{cases} \tag{3}$$

Low kSQI indicates low frequency noise such as baseline wonder, Gaussian (thermal observation) noise and high frequency sinusoidal noise (power-line interference)[3].

Frequency bounding (SQI_f)

fSQI measured whether the instantaneous heart rate fell within a valid physiological range, taken to be between 30 to 90 beats/min (see **??** (a), (b) and (c)). Instantaneous heart rate was estimated by the time difference between consecutive R-peaks detected using the Pan-Tompkins QRS detector. Given IHR_i as the instantaneous heart rate of the $\mathrm{k^{th}}$ beat, fSQI was defined as:

$$\mathrm{SQI_f}(k) = \begin{cases} 1 & \text{if} \quad 30 \leq \mathrm{IHR}_i(k) \leq 90 \\ 0 & \text{otherwise} \end{cases} \tag{4}$$

Signal to noise ratio (SQI_{SNR})

snrSQI measured the strength of the signal carrying cardiac information. Given x as an ECG signal in a window w centred around the $\mathrm{k^{th}}$ beat and x_{filt} is the ECG signal x filtered with a band-pass filter with cut-off frequencies of 0.5 and 14 Hz, the signal-to-noise ratio was defined as:

$$\mathrm{SNR}(k) = 10\log_{10}\frac{\mathrm{VAR}(x_{\mathrm{filt}})}{\mathrm{VAR}(x - x_{\mathrm{filt}})} \tag{5}$$

where $\mathrm{Var}(x)$ computes the variance of x. A high SNR implies a good quality for the signal. Therefore, snrSQI was defined as:

$$\mathrm{SQI}_{\mathrm{SNR}}(k) = \begin{cases} 1 \quad \text{if} \quad \mathrm{SNR}(k) \geq 10 \\ \frac{\mathrm{SNR}(k)}{10} \quad \text{if} \quad 0 \leq \mathrm{SNR}(k) \leq 10 \\ 0 \quad \text{otherwise} \end{cases} \tag{6}$$

Combined SQI (SQI_{ECG})

The combined ECG SQI, $\mathrm{SQI}_{\mathrm{ECG}}$, of the kth beat was calculated as:

$$\mathrm{SQI}_{\mathrm{ECG}}(k) = \begin{cases} \mathrm{SQI}_{\mathrm{b}}(k) \times \mathrm{SQI}_{\mathrm{f}}(k) \times \mathrm{SQI}_{\mathrm{SNR}}(k) & \text{if} \quad \mathrm{SQI}_{\mathrm{k}}(k) = 1 \\ \mathrm{SQI}_{\mathrm{b}}(k) \times \mathrm{SQI}_{\mathrm{f}}(k) \times \mathrm{SQI}_{\mathrm{SNR}}(k) \times \eta & \text{if} \quad \mathrm{SQI}_{\mathrm{k}}(k) = 0 \end{cases} \tag{7}$$

where η was a coefficient denoting the presence of statistical noise which was determined to be present whenever the kurtosis of the signal was lower that 5. The value of η was chosen to be 0.9.

Supplementary material E

PPG SQI

For PAT estimation, an evaluation of the signal quality of the PPG waveform was required. To assess the signal quality of the PPG, we followed the work of Villarroel[7]. Four different signal quality metrics were employed and combined together to provide a beat-by-beat SQI value. These metrics included: An assessment of clipping in the signal (SQI_c); sharp changes in amplitude (SQI_a); IHR outside of the physiological bounds (SQI_f); the deviation of beats from running-average window template (SQI_{DTW}). All quality metrics were combined to compute a signal quality estimate, (SQI_{PPG}), for each distal fiducial point.

Clipping detection (SQI_c)

Clipping generally occurs as a result of motion artefacts. Signal clipping can be detected when the derivative crosses a given threshold. Given that $N_{length}(k)$ is the length of the kth beat and $N_{clipped}(k)$ is the amount of the derivative of the kth beat that crosses a clipping threshold of 0.1, the cSQI of the kth beat was set to 0 when more than one-third of the derivative was clipped:

$$SQI_c(k) = \begin{cases} 0 & \text{if} \quad N_{clipped}(k)/N_{length}(k) > 1/3 \\ 1 & \text{otherwise} \end{cases} \tag{8}$$

Amplitude thresholding (SQI_a)

Amplitude thresholding was performed to determine whether the amplitude of each beat remained within three standard deviations, σ_w, from the mean μ_w of the window w. The statistics were calculated locally for each moving window w. The aSQI of the kth beat was set to 0 if part of the beat was outside the valid range:

$$SQI_a(k) = \begin{cases} 0 & \text{if} \quad \exists i \in b_k \qquad \mu_w - 3\times\sigma_w < \text{PPG}(i) \quad > \mu_w + 3\times\sigma_w \quad \text{or} \\ 1 & \text{otherwise} \end{cases} \tag{9}$$

where b_k is the location of the entire k^{th} beat (i.e. not just the fiducial point).

Frequency bounding (SQI_f)

Frequency bounding determined whether the instantaneous heart rate fell within a valid physiological range, taken to be between 30 to 90 beats/min. Instantaneous heart rate was estimated by the time difference between beat peaks detected using the BSSF algorithm. Given IHR_i as the instantaneous heart rate of the kth beat, fSQI was defined as:

$$SQI_f(k) = \begin{cases} 1 & \text{if} \quad 30 \le IHR_i(k) \le 90 \\ 0 & \text{otherwise} \end{cases} \tag{10}$$

Dynamic Time Warping (SQI_{DTW})

Dynamic time warping (DTW) is a time series technique used to determine a distance (or a degree of similarity) between two given time series based on the best possible alignment between the two. Importantly, DTW is able to compute a metric of similarity for a time series whose period changes over time. As a result, DTW is ideal for computing how similar PPG beats are even when the heart rate changes. The algorithm starts by constructing a beat template over a 20 second window by averaging the beats within the window[7]. Once a template has been computed for a given window, DTW is computed between each beat within the time window and the template.

Given a PPG beat $X = \{x_1, x_2, ..., x_N\}$ of length N within the current window and the template $Y = \{y_1, y_2, ..., y_M\}$ of length M. DTW finds the optimum warping path, $p = \{p_1, p_2, ..., p_L\}$ of length L, between the beat and the template. To do this a cost function, $c(x_i, y_j)$ is constructed defining the local distance between the current beat X at location i and the template Y at location j. The cost has a small value if x_i and y_i are similar to each other, and a large value if they are different. The DTW can then be computed by determining the path, p that minimises this cost as:

$$DTW(X,Y) = \underset{p}{\text{argmin}} \sum_{l=1}^{L} c(x_{nl}, y_{ml}) \tag{11}$$

The classical DTW algorithm is computationally expensive as it needs to evaluate every possible warping path in order to obtain an optimal alignment. Therefore, multi-scale dynamic time warping for pulsatile signals was performed to refine the

search space for the optimum alignment between the two time series. Multi-scale DTW is described in more detail together with its mathematical derivations in[7].

A good-quality beat will have a low value of DTW such that it has a similar shape to the average beat in the time window. dtwSQI was defined as:

$$\mathrm{SQI}_{\mathrm{DTW}}(k) = 1 - \frac{DTW(X_k, Y_k)}{100} \tag{12}$$

Combined SQI (SQI_{PPG})

The combined PPG SQI, $\mathrm{SQI}_{\mathrm{PPG}}$, of the kth beat was derived by multiplying all the SQI metrics together:

$$\mathrm{SQI}_{\mathrm{PPG}}(k) = \mathrm{SQI}_{\mathrm{c}}(k) \times \mathrm{SQI}_{\mathrm{a}}(k) \times \mathrm{SQI}_{\mathrm{f}}(k) \times \mathrm{SQI}_{\mathrm{DTW}}(k) \tag{13}$$

Supplementary material F

Resampling of ICG to 1kHz

An ICG signal recorded at 200 Hz provides a time resolution of 5 ms for detection of the B-point (point of aortic valve opening) or C-point (maximum of ICG pulse). This may lead to considerable errors in PEP estimates in this dataset. Many bioimpedance monitors will sample at 1 kHz to reduce this error[8]. This section compares the PEP estimates used in this paper (from the original 200 Hz ICG signal) to PEP estimates from an upsampled ICG at 1 kHz via cubic spline interpolation. Figure 11 shows the difference in PEP values from the two ICG signals for all volunteers in the dataset. Figure 12 compares the two signals across the entire dataset. The differences were low (mean difference = 3 ms), there was a bias towards the 200 Hz ICG over estimating the PEP signal compared to the 1 kHz ICG. In general, the trends are consistent across the two signals. We conclude, therefore, that PEP estimates from the 200 Hz ICG were not a significant limitation to the results in this study.

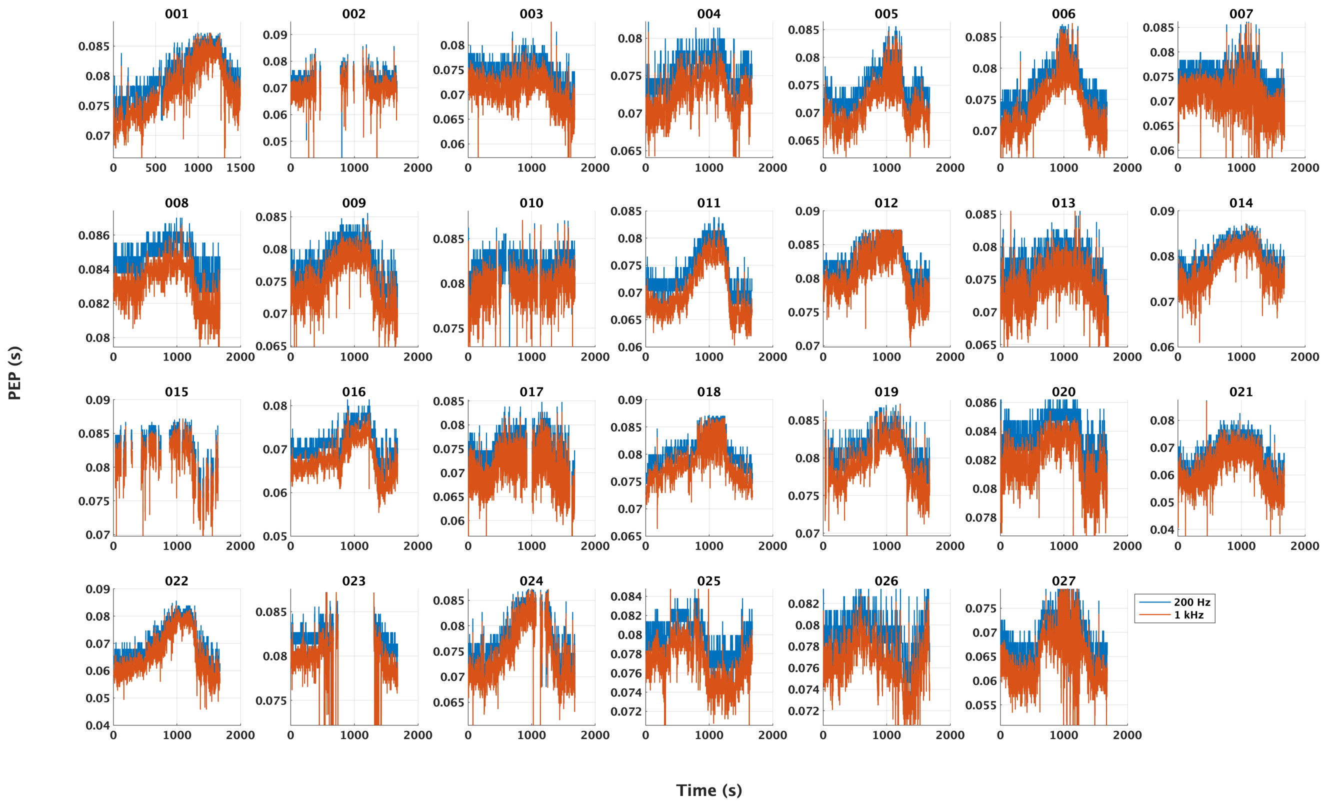

Figure 11. PEP estimates from the original 200 Hz ICG signal (blue) compared to PEP estimates from the 1 kHz upsampled ICG signal (red) for all volunteers in the dataset.

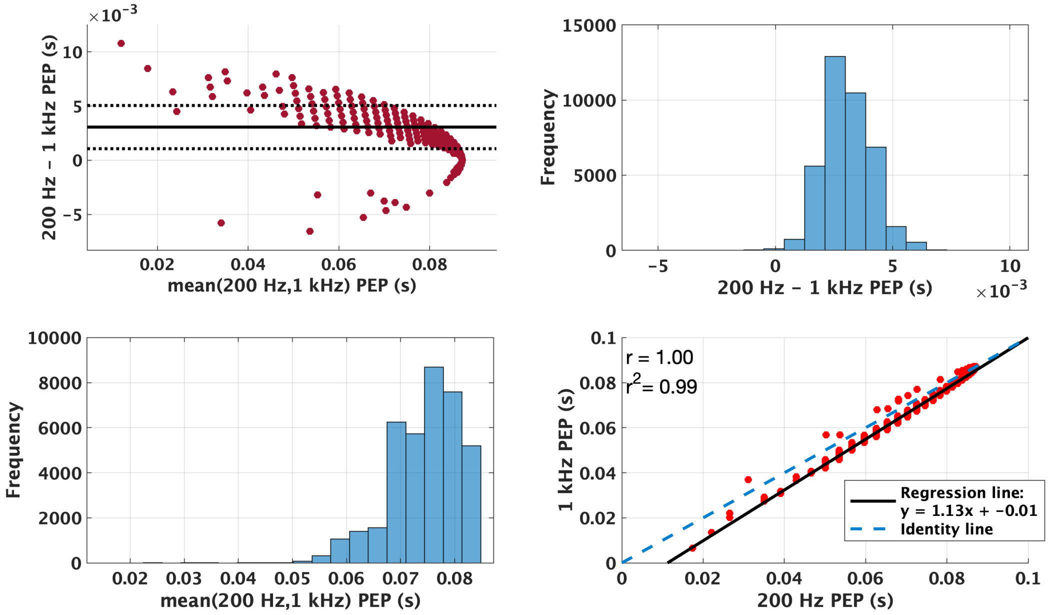

Figure 12. Comparison of PEP estimates from the two ICG signals (original 200 Hz ICG and upsampled 1 kHz ICG). The errors were low with a slight bias where PEP estimates from the 200 Hz signal were generally higher.

Supplementary material G

Changes in the PPG morphology

Figure 13 shows the PPG morphology from the same volunteer for typical pulses at (a) rest, (b) max phenylephrine infusion, and (c) washout. Wave reflections from points of impedance mismatch cause significant changes in the PPG morphology especially at the peak, making the peak an unreliable marker of arrival of the primary pressure wave in this dataset. As a result the foot of the PPG was used to define the point of arrival, this was defined using the intersecting tangents method.

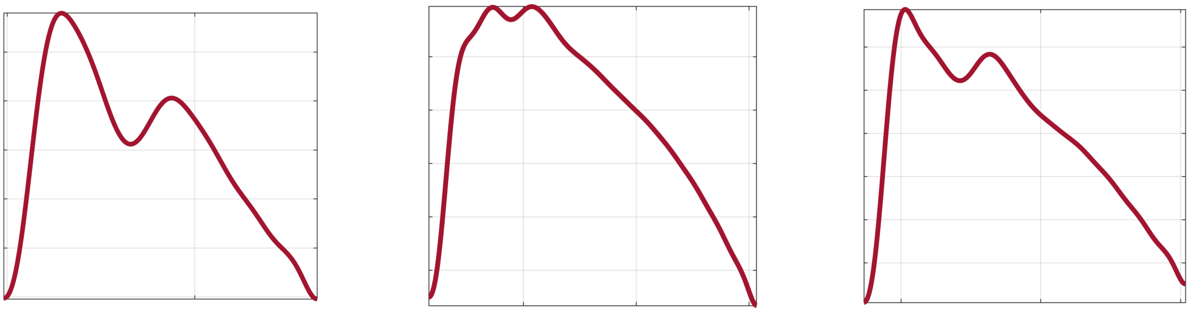

(a) Rest period

(b) Max infusion

(c) Washout period

Figure 13. Typical PPG morphology from the same volunteer at (a) rest, (b) max infusion, (c) washout.

References

1. Julien, C. The enigma of Mayer waves: facts and models. *Cardiovasc. research* **70**, 12–21 (2006).

2. Stergiou, G. S. *et al.* A universal standard for the validation of blood pressure measuring devices: Association for the Advancement of Medical Instrumentation/European Society of Hypertension/International Organization for Standardization (AAMI/ESH/ISO) Collaboration Statement. *Hypertension* **71**, 368–374, DOI: 10.1161/HYPERTENSIONAHA.117.10237 (2018).

3. Li, Q., Mark, R. G. & Clifford, G. D. Robust heart rate estimation from multiple asynchronous noisy sources using signal quality indices and a Kalman filter. *Physiol. Meas.* **29**, 15–32, DOI: 10.1088/0967-3334/29/1/002 (2008).

4. Pan, J. & Tompkins, W. J. A Real-Time QRS Detection Algorithm. *IEEE Transactions on Biomed. Eng.* **BME-32**, 230–236, DOI: 10.1109/TBME.1985.325532 (1985).

5. Zong, W., Moody, G. B. & Jiang, D. A robust open-source algorithm to detect onset and duration of QRS complexes. In *Computers in Cardiology*, vol. 30, 737–740, DOI: 10.1109/cic.2003.1291261 (IEEE, 2003).

6. ANSI/AAMI-EC57:1998. Testing and reporting performance results of cardiac rhythm and ST segment measurement algorithms. *Assoc. for Adv. Med. Instrumentation* **1998**, 36, DOI: 10.5594/j17740 (1999).

7. Villarroel, M. *Non-Contact Vital Sign Monitoring in the Clinic*. Ph.D. thesis, Oxford University (2017). DOI: 10.1109/FG.2017.43.

8. Sheikh, S.-a. A. *et al.* An open-source automated algorithm for removal of noisy beats for accurate impedance cardiogram analysis. *Physiol. Meas.* DOI: 10.1088/1361-6579/ab9b71 (2020).
